# Siphonous green macroalgae with contrasting capacities for the energy-dependent quenching, qE, rely on different photoprotective mechanisms

**DOI:** 10.1007/s11120-026-01225-1

**Published:** 2026-06-26

**Authors:** Heta Mattila, Vesa Havurinne, Paulo Cartaxana, Sónia Cruz

**Affiliations:** 1 CESAM–Centre for Environmental and Marine Studies, Department of Biology, University of Aveiro, Aveiro, Portugal; 2 Molecular Plant Biology, Department of Life Technologies, University of Turku, Turku, Finland

**Keywords:** Cyclic electron flow, Dynamic light, Photodamage, PTOX, Repair cycle, Ulvophyceae

## Abstract

Bryopsidales green macroalgae can induce non-photochemical quenching (NPQ) only slowly, presumably due to the lack of the proton gradient-induced component (qE) of NPQ and xanthophyll cycle. Here, two morphologically rather similar siphonous macroalgae, a Bryopsidales alga *Bryopsis* sp. and a Dasycladales alga *Acetabularia acetabulum*, latter of which is capable of qE, were given high light treatments with both constant and fluctuating intensity. No differences in the rate of photoinhibition of Photosystem II (PSII), estimated with the chlorophyll *a* fluorescence parameter F_V_/F_M_, in the absence or presence of lincomycin, were observed between constant and fluctuating light, nor between the two algae. *Bryopsis* sp. showed slower PSII recovery than *A. acetabulum*, possibly reflecting a regulatory response rather than increased oxidative stress, as the recovery rates increased with increasing amounts of PSII photoinhibition in both algae. In *Bryopsis* sp., however, high light treatments led to decreased electron transfer rates, estimated by both chlorophyll *a* fluorescence and net oxygen production, whereas a stimulation was observed in *A. acetabulum*. Nigericin, which prevents the formation of qE, increased photoinhibition in *A. acetabulum* but not in *Bryopsis* sp. Microoxic conditions as well as inhibitors of plastid terminal oxidase and mitochondrial respiration, on the other hand, enhanced photoinhibition only in *Bryopsis* sp., suggesting that, in the absence of qE, oxygen-dependent pathways (including flavodiiron proteins) are important for photoprotection. Near-infra-red absorption measurements suggest decreased Photosystem I (PSI) donor side limitation in *Bryopsis* sp., compared to *A. acetabulum*, and a lower capacity to keep P700 oxidised.

**Supplementary Information:**

The online version contains supplementary material available at 10.1007/s11120-026-01225-1.

## Introduction

Light damages the photosynthetic machinery of plants, algae and cyanobacteria and promotes the production of reactive oxygen species (ROS). Photosystem II (PSII) is particularly sensitive to photodamage. Multiple mechanisms may contribute to its damage: direct absorption of radiation (mostly UV and blue light) by the manganese ions of the oxygen evolving complex (Hakala et al. 2005; Ohnishi et al. 2005), oxidation by singlet oxygen (Vass 2012; Mattila et al. 2022a) and oxidation by long-lived PSII reaction centre radicals P680^+^ or TyrZ^•^ (Jegerschöld et al. 1990; Mattila et al. 2022a). The rate constant of PSII photoinhibition is directly proportional to the intensity of light (Tyystjärvi and Aro 1996), but net damage only accumulates if the concurrent PSII repair cycle fails to keep up with the rate of the damage, for example, under high light (for a recent review of PSII repair, see Su et al. 2024). In addition, in many organisms, the repair cycle is sensitive to ROS (Nishiyama et al. 2001; Toriu et al. 2023).

Non-photochemical quenching (NPQ) of excitation energy is a ubiquitous photoprotective mechanism of photosynthetic organisms. In the green microalga *Chlamydomonas reinhardtii*, NPQ largely depends on the light-harvesting complex stress-related (LHCSR) proteins (Peers et al. 2009; Bonente et al. 2011; Tian et al. 2019; Perozeni et al. 2020). LHCSR-dependent NPQ is rapidly activated by the protonation of the thylakoid lumen and is thus called energy-dependent quenching (qE). In plants and some green algae, low luminal pH also activates the xanthophyll cycle (conversion of violaxanthin to zeaxanthin), which further enhances NPQ (Quaas et al. 2015; Girolomoni et al. 2020). NPQ diminishes ROS production (Girolomoni et al. 2017; Barera et al. 2021; Troiano et al. 2021) as well as photoinhibition of PSII (Jahns et al. 2000; Tyystjärvi 2013; Barera et al. 2021). Consequently, mutants deficient in NPQ are often sensitive to high and fluctuating light (Külheim et al. 2002; Peers et al. 2009; Cantrell and Peers 2017; Steen et al. 2022).

However, a group of green macroalgae, Bryopsidales, appears to lack genes for the LHCSR proteins and for the Photosystem II 22 kDa (PSBS) protein (Handrich et al. 2017; Iha et al. 2021; Xu et al. 2025), which is the sole pH-sensor of NPQ in plants. Consequently, in Bryopsidales, NPQ is induced (and relaxed) slowly and occurs independently of a proton gradient (Christa et al. 2017). Furthermore, only minimal zeaxanthin accumulation is observed during dark to light transitions in Bryopsidales algae (Raniello et al. 2006; Christa et al. 2017). In *C. reinhardtii*, LHCSR proteins are not expressed under low light (Peers et al. 2009). In Bryopsidales, however, the absences of fast NPQ induction and violaxanthin to zeaxanthin conversion upon high light illumination are observed in algae cultured either under high or low light (Christa et al. 2017).

State transitions balance excitation between PSII and Photosystem I (PSI); reduction of the plastoquinone pool induces phosphorylation, detachment and possibly movement of a part of the PSII antenna to serve PSI (state 1 to state 2 transition; Delosme et al. 1996; Depège et al. 2003; Huang et al. 2021). In green algae, state transitions are involved in photoprotection and double mutants deficient in NPQ and state transitions are more sensitive to PSII photoinhibition and produce more ROS than the respective single mutants (e.g., Cardol et al. 2009; Allorent et al. 2013; Roach et al. 2015). Interestingly, the studied Bryopsidales algae have lost, in addition to qE, the capacity for state transitions (Havurinne et al. 2025).

Besides NPQ and state transitions, auxiliary electron transfer pathways contribute to photoprotection, for example, by reducing ROS production. Plastid terminal oxidase (PTOX) oxidizes the PQ pool by reducing oxygen (for a review, see Nawrocki et al. 2015). Under constant light, PTOX may have a minor role (Bonente et al. 2012; Peltier et al. 2024), but under fluctuating light, a *C. reinhardtii* mutant deficient in PTOX activity grows slowly, possibly due to over-reduction of the PQ pool during dark periods (Nawrocki et al. 2019).

Flavodiiron proteins mediate electron transfer from PSI to oxygen (safely producing water) and are especially important for the photoprotection of PSI (Inoue 1986; Tiwari et al. 2016) in fluctuating light (Chaux et al. 2017a; Jokel et al. 2018) but have also been shown to protect PSII (Zhang et al. 2009; Bersanini et al. 2017). *C. reinhardtii* mutants lacking flavodiiron proteins also show increased PSI-derived ROS production (Pfleger et al. 2024).

Cyclic electron transport from PSI back to the PQ pool produces extra ATP, possibly powering PSII repair (Huang et al. 2018), besides protecting PSI in fluctuating light (Jans et al. 2008; Chaux et al. 2017b; Jokel et al. 2018; Nikkanen et al. 2020). In *C. reinhardtii*, the cyclic electron transfer occurs via the type II NAD(P)H dehydrogenase (NDA-2) and/or the proton gradient regulation 5 (PGR5)/PGR5-like (PGRL1) routes (Desplats et al. 2009; Patil et al. 2022). Although some algae may possess the type I NAD(P)H dehydrogenase complex (NDH-1), prevalent in cyanobacteria and plants (for a review, see Ma et al. 2021), the genes are missing from the chloroplast genomes of the studied Bryopsidales and Ulvophyceaen algae (e.g., Melton et al. 2015; Leliaert and Lopez-Bautista 2015). Thus, these algae presumably rely on the NDA-2 cyclic route. The PGR5/PGRL route may affect cyclic electron transfer by regulating ATP synthase activity, rather than directly transferring electrons to the PQ pool (Nandha et al. 2007; Nikkanen et al. 2024). Regardless, it has been suggested that cyclic electron transfer is especially important under anoxic conditions, where flavodiiron proteins (and other oxygen-dependent pathways) cannot function (Tolleter et al. 2011; Alric 2014; Godaux et al. 2015). Accordingly, *C. reinhardtii* upregulates the PGR5 pathway under anoxia (Alric 2014; Godaux et al. 2015).

Finally, mitochondrial respiration and exchange of reducing power and energy equivalents between chloroplasts and mitochondria (via malate shuttles, an ADP/ATP translocator and/or a triose phosphate transporter; Lemaire et al. 1988; for a review, see Burlacot et al. 2019) are important, especially under high light (Kaye et al. 2019; Huang et al. 2023; Peltier et al. 2024).

Many Bryopsidales algae inhabit harsh environments, such as tidal pools, and yet, they seem to lack both qE and state transitions. Bryopsidales contain the unique carotenoids siphonaxanthin and siphonein (Ricketts 1971), which are sometimes suggested to contribute to their survival (e.g., Agostini et al. 2025). Here, we hypothesize that Bryopsidales utilize compensatory photoprotective mechanisms to survive. Thus, we compared two optically rather similar siphonous green macroalgae (Fig. S1): the qE-deficient Bryopsidales alga *Bryopsis* sp., and a “true Ulvophyte” (for a recent phylogenetic analysis of the group Ulvophyceae, see Hou et al. 2022), the Dasycladales alga *Acetabularia acetabulum*, which has qE capacity. Based on chlorophyll *a* fluorescence measurements, PSII in *Bryopsis* sp. is not more vulnerable to fluctuating light than it is to constant light, similarly as in *A. acetabulum*. However, the two species exhibited differing vulnerabilities to inhibitors of different photoprotective pathways suggesting that in the absence of qE, Bryopsidales algae rely more on other available photoprotective mechanisms.

## Materials and methods

### Algal species and culture conditions

*A. acetabulum* (strain DI1 isolated by Diedrik Menzel) and *Bryopsis* sp. (previously *B. plumosa*; KU-0990; obtained from Kobe University Macroalgal Culture Collection, Japan) were grown under laboratory conditions (Fig. S1A and B) at 20–22 °C, in the photosynthetic photon flux density (PPFD) of 40–80 µmol m^− 2^ s^− 1^, under 12/12 hour day/night cycle, in modified f/2 medium, without silica, prepared in 35 ppt artificial seawater (ASW; Red Sea salt, Red Sea Europe, France), as previously described (Havurinne and Tyystjärvi 2020; Cartaxana et al. 2023).

### High light treatments

Intact algal cells were placed in artificial seawater, supplemented, when indicated, either with 10 µM antimycin A (AA), 6 mM glucose, 8 U/mL glucose oxidase and 800 U/mL catalase (to induce anaerobicity), 1 mM dithiothreitol (DTT), 60 µM nigericin (NIG), 10 µM oligomycin (OM), 1 mM propyl gallate (PG) or 0.2 mM polymyxin B (PMB). Stock solutions of glucose, glucose oxidase, catalase, DTT and PMB were prepared in distilled water, those of AA, PG and NIG in ethanol and that of OM in dimethyl sulfoxide. The algae were let to acclimate for 20 min in the dark, unless otherwise specified, after which they were illuminated for 50 min with white or blue high light (for the spectra, see Fig. S2A) of constant or fluctuating intensity, as specified below and in the respective figure legends. The PPFD of the constant illumination was 500 µmol m^− 2^ s^− 1^. For the fluctuating light treatment, light intensity gradually increased for five min (from 0 to the PPFD of 1000 µmol m^− 2^ s^− 1^) and then again decreased for five min; the cycle was repeated five times (Fig. S2B), to mimic natural conditions where light intensity often changes gradually due to e.g., changing cloud coverage and wave movements. The cumulative (total) PPFD was the same in both light treatments. Light intensity was measured with a wavelength-calibrated ULM-500 Universal Light Meter with a planar sensor (Walz, Germany) in air. All the treatments were performed at room temperature (~ 20 °C).

Actual oxygen concentrations in the presence of glucose, glucose oxidase and catalase were estimated with an oxygen electrode (Oxytherm + P; Hansatech Instruments; UK) at 20 °C in a 2 mL chamber. Measurements were taken during illumination of algal samples with the white build-in LED of the oxygen electrode system. The chamber was left open for the duration of the experiment, to mimic the conditions under which the above-described high light experiments were carried out.

### High performance liquid chromatography

For analyses of the xanthophyll cycle pigments, algae were given the above-described constant and fluctuating light treatments with the Reef Pulsar SPS-8 LED light source (see Fig. S2A for the spectrum; Tropical Marine Centre; UK) in plain artificial seawater. After 20 min in the dark, and after five, 10 (only fluctuating light), 45 and 50 (only fluctuating light) min in the light and after subsequent five min in the dark (only constant light), the algal samples were immediately frozen in liquid nitrogen and stored at -80 °C until freeze-dried. In the case of *Bryopsis* sp., only the time points at 20 min in the dark, and 45 and 50 min in fluctuating light were sampled. Pigments were then quantified by high performance liquid chromatography (HPLC), as described by Marques et al. (2021). Shortly, freeze-dried samples were sonicated for one min in ice cold 95% buffered methanol (2% ammonium acetate) and incubated for 20 min in the dark at -20 °C for pigment extraction. Filtered extracts (0.2 μm PTFE filters) were injected into the HPLC system (Prominence-i LC 2030 C; Shimadzu, Kyoto, Japan) equipped with a Supelcosil C18 column (dimensions: 250 × 4.6 mm; particle size: 5 μm; Sigma-Aldrich, St Louis, MO, USA). Photosynthetic pigments were identified based on their retention times and absorption spectra. Calibration curves were constructed with five dilutions of pure pigment standards from DHI (Hørsolm, Denmark). Pigments were expressed in mg g DW^− 1^ (dry weight). The operation of the xanthophyll cycle in *A. acetabulum*, comprising of the sequential de-epoxidation of the pigments violaxanthin (Viola) to antheraxanthin (Anth) and zeaxanthin (Zea), was quantified by calculating the de-epoxidation state (DES) as: DES = ([Zea] + 0.5×[Anth])/([Zea]+[Anth]+[Viola]).

### Chlorophyll *a* fluorescence

#### Fluorescence kinetics

Pulse amplitude modulated (PAM) chlorophyll *a* fluorescence kinetics under the above-described constant and fluctuating light treatments (under blue light; for the light spectrum, see Fig. S2A) were measured with the Imaging-PAM fluorometer (MINI version; Walz, Germany), unless otherwise stated, in plain artificial seawater as well as in the presence of either AA, DTT, NIG, OM, PG or PMB, or under anoxia. Frequency of the measuring flashes (setting 2) was 1 Hz. F_V_/F_M_ (=(F_M_-F_O_)/F_M_), where F_M_ is the maximum fluorescence (during a saturating light pulse; 600 ms, setting 9; PPFD ~ 9 000 µmol m^− 2^ s^− 1^) and F_O_ minimum fluorescence (only measuring flashes on) of a dark-acclimated sample, was measured after 20 min in the dark. During the illumination, saturating light pulses were fired every min and during the subsequent darkness, every one to five min, to calculate NPQ ((F_M_-F_M_’)/F_M_’), PSII “operational efficiency” ((F_M_’-F’)/F_M_’) and photochemical quenching qP ((F_M_’-F’)/(F_M_’-F_O_’), where F’ is fluorescence yield under incident illumination (usually measured prior to a saturating light pulse) and F_M_’ is the maximum fluorescence (during a saturating light pulse) under illumination. F_O_’ was estimated as F_O_/(F_V_/F_M_+F_O_/F_M_’), according to Oxborough and Baker (1997). For analysis of the data, small sections from individual algal cells were selected.

#### Rapid light response curves

Relative electron transport rates (rETR) were probed by recording rapid light response curves with the Imaging-PAM before and after the above-described constant and fluctuating light treatments with white light using the Reef Pulsar SPS-8 (see Fig. S2A for the spectrum), in plain artificial seawater as well as in the presence of PG or under anoxia or both. Algae were acclimated to low light (PPFD 10–20 µmol m^− 2^ s^− 1^) for 20 min and then illuminated with increasing intensities of blue light (PPFDs 4, 17, 39, 57, 84, 138, 178, 240, 309, 373, 448, 540 and 645 µmol m^− 2^ s^− 1^), for 60 s each, after which a saturating light pulse was fired to calculate rETR and NPQ; rETR was calculated as (F_M_’-F’)/F_M_’ × 0.5 × 0.84 × PPFD (note that the PSII light absorption cross-section was not measured from the algae and therefore the rates of PSII electron transfer cannot be directly compared between the algae). To obtain values of alpha (initial slope of the light response curve), maximum rate of relative electron transfer (rETR_MAX_) and minimum saturating irradiance (I_K_), the curves were fitted to the model of Eilers and Peeters (1988) in Microsoft Excel.

#### Photoinhibition of PSII

To probe the amount of PSII damage and repair, algae were given the above-described constant and fluctuating light treatments under white light with the Reef Pulsar SPS-8 (see Fig. S2A for the spectrum) and dark controls of similar lengths, in plain artificial seawater as well as in the presence of either AA, DTT, NIG, OM, PG or PMB, or under anoxia. In addition, experiments were also conducted in the presence of 10 mM lincomycin (LM); LM was dissolved in 20 mL of artificial seawater, where the algae were incubated overnight in darkness. Before the light treatments, algae were removed from the LM solution and placed in fresh artificial seawater. After the high light treatments (without LM), two-hour recovery was performed under low light (white halogen lamps; PPFD 10–20 µmol m^− 2^ s^− 1^). To measure PSII activity, F_V_/F_M_ values were recorded with the Imaging-PAM as described above, after 20 min in the dark. A decrease in the F_V_/F_M_ value (in respect to the values before adding the chemicals; or further 20 min dark incubation in the absence of the additions) during a 50 min incubation in the dark in the presence of the above-mentioned chemicals was added to the final values, to compensate for any dark-induced effects of the chemicals.

#### F_O_ rise

Post-illumination rise in chlorophyll *a* fluorescence (F_O_ rise) yield was measured with Junior-PAM (Walz) from algae dark acclimated in artificial seawater for 20 min. The F_O_ rise protocol was similar to the protocol described by Mattila et al. (2022b). Briefly, the algae were illuminated with far-red light (PFD 50 µmol m^− 2^ s^− 1^; see Havurinne et al. 2025 for the spectrum) for five min after which the far-red light was switched off, and only the weak blue measuring light was kept on. Before, during and after the far-red illumination, saturating light pulses (800 ms) were fired to check NPQ levels. The measuring light frequency was five Hz using the intensity setting three, and the saturating light pulse intensity was set to 12 (maximum; PPFD ~ 7000 µmol m^− 2^ s^− 1^).

### Photoinhibition from isolated thylakoid membranes

To verify the chlorophyll *a* fluorescence-based estimations of PSII photoinhibition, thylakoid membranes of *Bryopsis* sp. were isolated by breaking three batches of cells by grinding in ice-cold mortar, according to Hakala et al. (2005). Isolations were conducted before and immediately after (i.e., the algae were not dark acclimated after the illumination) the above-described fluctuating light treatment under white light with the Reef Pulsar SPS-8 (see Fig. S2A for the spectrum) in plain artificial seawater. Maximum oxygen evolution activity of PSII was measured with an oxygen electrode (Oxytherm + P; Hansatech Instruments) immediately after the isolations, as follows. The thylakoids were dissolved in a buffer containing 40 mM Hepes-KOH (pH 7.4), 330 mM sorbitol, 1 M betaine monohydrate, 5 mM MgCl_2_, 5 mM NaCl, 1 mM KH_2_PO_4_, 5 mM NH_4_Cl, 0.5 mM 2,6-dichloro-1,4-benzoquinone (DCBQ) and 0.5 mM Potassium hexacyanoferrate(III), placed into the 2 mL closed chamber of the oxygen electrode and maximum (light-saturated) PSII oxygen evolution activity was measured under saturating white light (PPFD 4000 µmol m^− 2^ s^− 1^) at 20 °C. The oxygen evolution rates were quantified by calculating a linear regression at 40–90 s after switching on the saturating light; the rate of oxygen consumption under dark prior to switching on the light was subtracted from the final results. The rates were finally quantified based on chlorophyll content of the thylakoid sample; chlorophyll contents were quantified with a spectrophotometer according to Porra et al. (1989), after ~ one hour extraction in acetone in the dark and subsequent centrifugation (a white pellet, according to visual inspection, was obtained indicating that chlorophylls were fully extracted).

### Oxygen evolution by intact cells

To measure net oxygen production, intact algae were given the above-described constant light treatments using the white build-in LEDs of the oxygen electrode system (Oxytherm + P; Hansatech Instruments) in plain artificial seawater, at 20 °C, in a two mL closed chamber. Rates of oxygen consumption/evolution were quantified by calculating instantaneous linear regression over one min during the whole measurement period. After the illumination, algal samples were gently dried with a tissue paper and weighed with an analytical scale and the oxygen production/consumption was quantified on the basis of the fresh weight of the sample.

### P700 kinetics

Redox kinetics of P700 (the reaction centre of PSI) were assayed by measuring near infra-red absorbance changes (at 830 nm, using 875 nm as a reference) with Dual-PAM-100 (Walz). Algae were placed in a regular spectrometer cuvette with two mL artificial seawater and dark-acclimated for 20 min in the absence or presence of AA or PMB. Algae were then given a saturating light pulse (PPFD ~ 13 000 µmol m^− 2^ s^− 1^; 800 ms) and illuminated for five min with increasing intensities of red light (PPFDs 50, 110, 215, 550 and 1080 µmol m^− 2^ s^− 1^; one min with each intensity). At the end of each light intensity, as well as after one and two min of subsequent darkness, a saturating light pulse was fired to calculate PSI yield ((P_M_’-P’)/P_M_) as well as donor (P’/P_M_) and acceptor side ((P_MAX_-P_M_’)/P_MAX_) limitation. Here, P_M_ and P_M_’ are P700 signals during a saturating light pulse in a dark acclimated sample and under illumination, respectively; P’ is the signal before the saturating light pulse (under incident illumination); P_MAX_ was defined as the highest of the measured P_M_ or P_M_’ signals of each sample. Due to a strong drift in the signal, the background level (obtained after a saturating light pulse) was subtracted from all the values (P_M_, P_M_’ and P’).

### Absorptance

Light absorptance was measured from intact cells using an integrating sphere (Labsphere, North Sutton, NH, USA). The samples were placed inside the sphere (in air), and a low voltage halogen lamp was used for a light source. Absorptance at 450–750 nm was measured with an STS-VIS spectrometer (Ocean Optics; USA). Calculation of absolute absorptance values was not attempted because the surface areas of the siphonous algal cells were difficult to determine. After the measurements, algal samples were gently dried with a tissue paper, weighed with an analytical scale and placed in N, N-dimethylformamide for four days in the dark. Chlorophylls were then quantified spectrophotometrically according to Porra et al. (1989).

### Imaging

Macro-imaging was conducted with a Canon EOS 7D MKII camera (Canon, Japan), equipped with a 100 mm f2.8 Ultra Macro APO 2:1 objective (Laowa, China) and a speedlight flash, with the settings ISO 100, f4.5 and 1/200s exposure time. Light microscopy was conducted with DMi1 inverted microscope, equipped with 5x, 10x and 20x PH1 objectives (HI Plan I; Leica Microsystems, Germany).

### Figures and statistical analyses

Figures were prepared with the ggplot2 v3.4.1 package of R (R Core Team 2021; Wickham 2016). T-tests (heteroscedastic) were calculated in Microsoft Excel, using a Bonferroni correction.

## Results

### *Bryopsis* sp. induced NPQ slowly and lacked light-induced violaxanthin to zeaxanthin conversion

Previous research suggest that Bryopsidales algae lack the fast, proton gradient induced components of NPQ. Therefore, we studied photoinhibition in a Bryopsidales alga (*Bryopsis* sp.) and, for comparison, in an Ulvophyceaen alga previously shown to possess qE-capacity (*A. acetabulum*). The algae were given 50 min high light treatments with either constant (PPFD of 500 µmol m^− 2^ s^− 1^) or gradually fluctuating (PPFDs between 0 and 1000 µmol m^− 2^ s^− 1^; see Fig. S2) intensity. To facilitate comparison between the constant and fluctuating light treatments, the total (cumulative) amount of light given was equal in both treatments.

*Bryopsis* sp. and *A. acetabulum* both form large, differentiated thalli comprising a single giant tubular (siphonous) cell, with similar diameters (see Fig. S1). First, we measured visible light absorptance from intact algal cells to estimate how comparable the given high light treatments were between *Bryopsis* sp. and *A. acetabulum*. In general, absorptances, normalized to fresh weight, were similar in *Bryopsis* sp. and *A. acetabulum* (Fig. S3A). However, *Bryopsis* sp. absorbed more light at green wavelengths (~ 500–600 nm), presumably due to the presence of the carotenoids siphonaxanthin and/or siphonein, explaining its brownish colour (see Fig. S1). *Bryopsis* sp. also contained ~ 30% more total chlorophylls (Fig. S3B) and ~ 43% more chlorophyll *b* (Fig. S3C) per fresh weight than *A. acetabulum*.

**Fig. 1:**
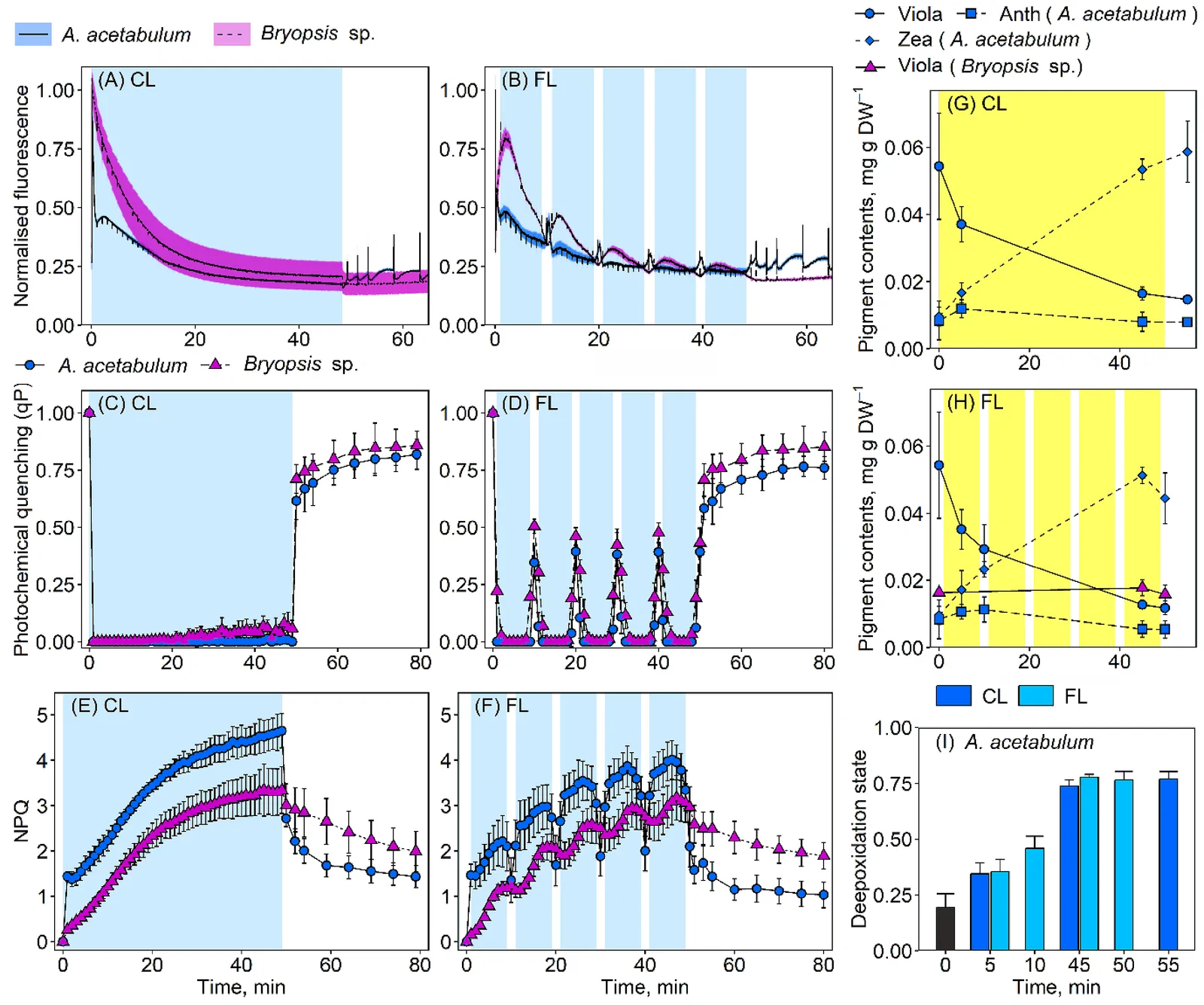
Chlorophyll *a* fluorescence kinetics and xanthophyll cycle pigments under constant and fluctuating light in *Acetabularia acetabulum* (continuous lines with blue shading or blue symbols) and *Bryopsis* sp. (dashed lines with purple shading or purple symbols). Dark-acclimated algae were illuminated with constant (**A**, **C**, **E** and **G**; PPFD 500 µmol m^− 2^ s^− 1^) or fluctuating (**B**, **D**, **F** and **H**; PPFD of 0 to 1000 µmol m^− 2^ s^− 1^; Fig. S2) blue (**A–F**; illustrated by the blue panels) or white light (G–I; illustrated by the yellow panels) and incubated subsequently in the dark. Chlorophyll *a* fluorescence kinetics during (**A**) constant and (**B**) fluctuating light, normalised to the starting values. Saturating light pulses were given before the illumination, every one min during the illuminations and every one to five min during the subsequent darkness. Photochemical quenching (qP) during (**C**) constant and (**D**) fluctuating light and non-photochemical quenching (NPQ) during (**E**) constant and (**F**) fluctuating light. Contents of the xanthophyll cycle pigments violaxanthin (Viola), antheraxanthin (Anth) and zeaxanthin (Zea), expressed on a dry weight (DW) basis, during (**G**) constant and (**H**) fluctuating white light in *A. acetabulum* and *Bryopsis* sp.; only violaxanthin was detected in *Bryopsis* sp. The same control sample (0-min) was used for both the light treatments. (**I**) De-epoxidation state of the xanthophyll cycle in *A. acetabulum*, during constant (CL) and fluctuating (FL) light and in darkness (the black bar), calculated based on (**G** and **H**). All the treatments were conducted at room temperature in artificial seawater. The data show averages and shaded areas (**A** and **B**) or error bars (**C–I**) standard deviations, calculated based on three to six biological replicates

Next, chlorophyll *a* fluorescence kinetics were recorded under constant and fluctuating high light in *Bryopsis* sp. and *A. acetabulum*. During both light treatments, fluorescence yield initially increased to a higher level in *Bryopsis* sp. than in *A. acetabulum*, but eventually decreased to similar, low levels, clearly below the original F_O_ levels (Figs. 1A and B and S4). Interestingly, after the constant illumination (Fig. 1A) and during the low light phases of the fluctuating light treatment (Fig. 1B), fluorescence behaved in a contrasting manner in the two algae; in *A. acetabulum*, fluorescence increased and in *Bryopsis* sp., it decreased, possibly due to relaxation of NPQ and oxidation of the plastoquinone pool, respectively.

During the constant illumination and high light phases of the fluctuating illumination, PSII “operational yield” (F_M_’-F’)/F_M_’ (Fig. S4) as well as photochemical quenching (qP; Fig. 1C and D) remained close to zero in both algae. During the low light phases of the fluctuating illumination, these PSII parameters increased very similarly in both algae, albeit to slightly higher levels in *Bryopsis* sp., especially at the beginning of the illumination. In *A. acetabulum*, however, the maximum values of (F_M_’-F’)/F_M_’ tended to remain constant or even slightly increase during the subsequent low light periods, while in *Bryopsis* sp. those values decreased over time (Fig. S4). A similar but weaker trend was visible in the qP parameters (Fig. 1C and D).

*A. acetabulum* rapidly induced NPQ at the start of the constant illumination and during the high light periods of the fluctuating illumination and also rapidly relaxed a major part of the induced NPQ during darkness or the low light periods (Fig. 1E and F). However, the rapid phase of NPQ induction at the start of the illumination was almost non-existent in *Bryopsis* sp., and only little relaxation of NPQ occurred during the low light periods (Fig. 1E and F). It should be noted, however, that the frequent (every min) use of high intensity saturating flashes may have prevented some relaxation of NPQ. Regardless, the maximum amount of NPQ induced by the constant and fluctuating light treatments were similar in both algae, although in both cases, *Bryopsis* sp. induced less NPQ than *A. acetabulum* (Fig. 1E and F).

The presence and absence of the xanthophyll cycle, in *A. acetabulum* and *Bryopsis* sp., respectively, were confirmed by measuring pigment contents with HPLC during the illuminations (Figs. 1G–I and S5). In *A. acetabulum*, violaxanthin was rapidly converted to zeaxanthin, upon the start of both constant and fluctuating light treatments and its contents further increased during the 50-min time courses of both illuminations (Fig. 1G and H). However, no recovery was observed in the violaxanthin or zeaxanthin amounts during the low light periods of the fluctuating light treatment (at five and 45 min) or at five min after switching off the constant light. The de-epoxidation state of the xanthophyll cycle increased very similarly during both constant and fluctuating light in *A. acetabulum* (Fig. 1I), in agreement with the similar NPQ amounts induced during these illuminations (Fig. 1E and F). As expected, almost no differences were observed in the violaxanthin contents in *Bryopsis* sp. under fluctuating light (Fig. 1H) and no antheraxanthin or zeaxanthin were detected (see Fig. S5G and H); the minor changes between the time-points in violaxanthin amounts were probably related to slightly differing average chlorophyll contents of the samples (Fig. S5). Small changes in the amounts of other pigments (9’-cis-neoxanthin, lutein and β-carotene in *A. acetabulum*, and siphonaxanthin, all-trans-neoxanthin, 9’-cis-neoxanthin, siphonein and α-carotene in *Bryopsis* sp.) also reflected the changes in average chlorophyll contents of the samples (Fig. S5).

### *Bryopsis* sp. displayed moderate PSII damage but slow PSII repair

**Fig. 2:**
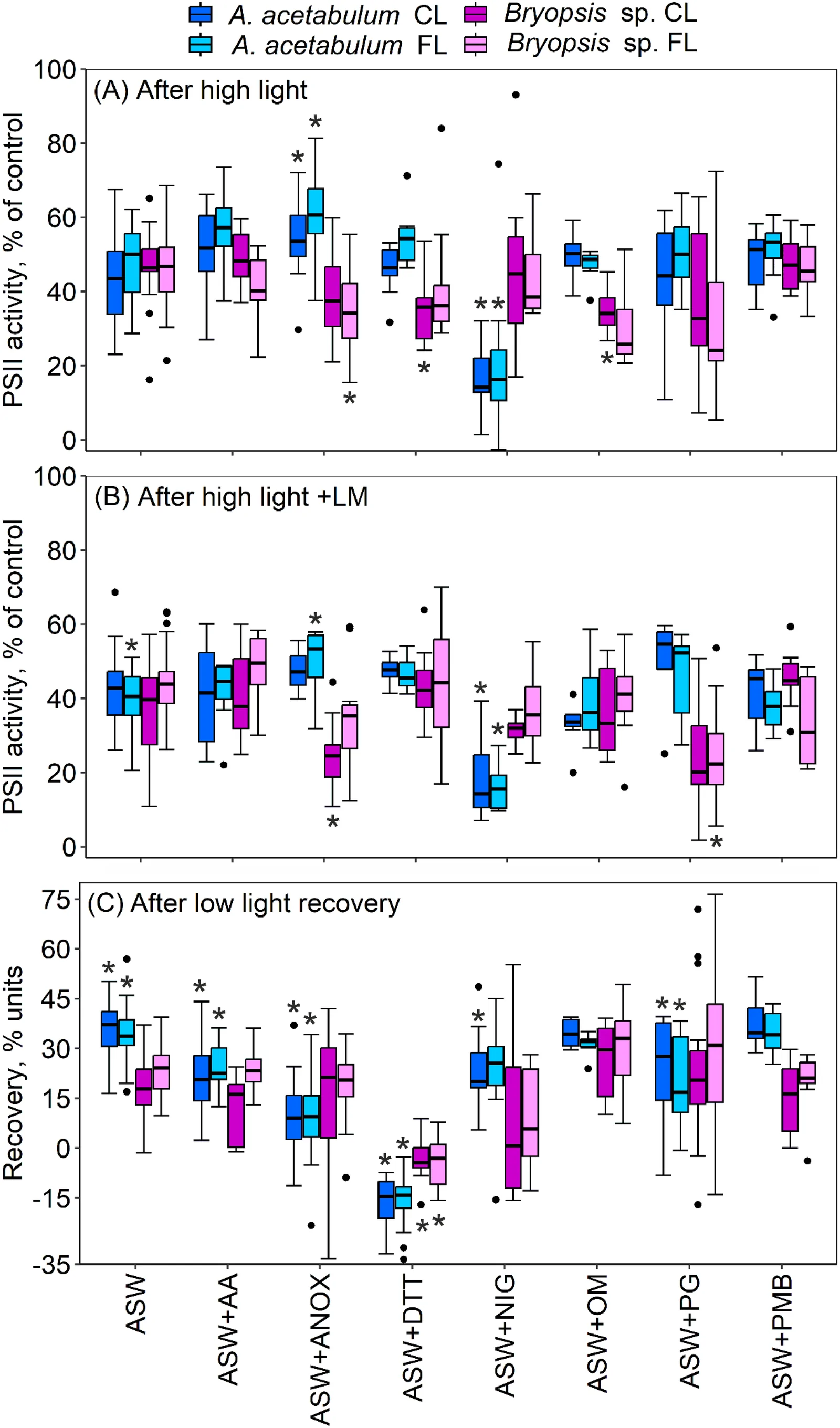
PSII photoinhibition and recovery in *Acetabularia acetabulum* (blue boxes) and *Bryopsis* sp. (purple boxes). The algae were illuminated for 50 min with constant (CL; PPFD 500 µmol m^− 2^ s^− 1^) or fluctuating (FL; PPFD 0 to 1000 µmol m^− 2^ s^− 1^; see Fig. S2) white light (**A**). (**B**) The high light treatments were also conducted with algae pre-incubated overnight in darkness in the presence of lincomycin (LM). (**C**) After the high light (without LM), algae were let to recover at low light (PPFD 10–20 µmol m^− 2^ s^− 1^) for two hours. All treatments were conducted at room temperature in artificial seawater (ASW) supplemented either with antimycin A (AA), glucose, glucose oxidase and catalase to induce anaerobicity (ANOX; Fig. S9), dithiothreitol (DTT), nigericin (NIG), oligomycin (OM), propyl gallate (PG) or polymyxin B (PMB), as indicated. PSII activity was probed with the chlorophyll *a* fluorescence parameter F_V_/F_M_, measured after 20 min in the dark, and quantified as the percentage of the initial (control) F_V_/F_M_ values (after addition of the chemicals but prior to the illumination; for the initial data, see Fig. S6). A decrease in the F_V_/F_M_ value during a 50-min dark incubation in the presence of the chemicals (Fig. S7) was taken into account. Recovery was quantified as the difference in the percentage units between high light and recovery ((F_V_/F_M_ recovery - F_V_/F_M_ high light)/(F_V_/F_M_ initial) x 100). The box plots show medians, 2nd and 3rd quartiles, error bars show minimum and maximum values and dots show outliers (> 1.5 times the interquartile range), calculated based on 6–28 biological replicates. The asterisks highlight chemical treatments that statistically significantly differ from their respective treatments in ASW alone, or in the case of ASW in (**B**), between LM-treated and non-treated samples, or in the case of ASW in (**C**), between *A. acetabulum* and *Bryopsis* sp. (see Tables S1–4 for details)

Next, to estimate the amount of PSII photoinhibition, *A. acetabulum* and *Bryopsis* sp. were again illuminated with constant or fluctuating light in the absence (Fig. 2A) or presence of lincomycin (Fig. 2B), an antibiotic that prevents the repair of damaged PSII units. In addition, recovery of the damaged PSII units was quantified after subsequent two-hour incubation at low light (PPFD of 10–20 µmol m^− 2^ s^− 1^) without lincomycin (Fig. 2C). All the treatments were also conducted in the presence of inhibitors of NPQ, cyclic, pseudo-cyclic or mitochondrial pathways, as specified below. PSII activity was probed with the chlorophyll *a* fluorescence parameter F_V_/F_M_ (after 20 min in the dark). Due to slightly lower starting F_V_/F_M_ values of *Bryopsis* sp., compared to those of *A. acetabulum* (Fig. S6), and because several of the inhibitors slightly lowered the F_V_/F_M_ values already in the dark (Fig. S7), results were normalized to their respective initial F_V_/F_M_ values (prior to the high light treatments but after addition of an inhibitor). In addition, a decrease in the F_V_/F_M_ value during a 50-min dark incubation (corresponding to the duration of the high light treatment) in the presence of the inhibitors was taken into account (see Fig. S6 for the original data and Material and methods for details).

When *A. acetabulum* and *Bryopsis* sp. were illuminated in plain artificial seawater without inhibitors, the F_V_/F_M_ values decreased very similarly in both algae and during both light treatments; no statistically significant differences were observed in the amount of PSII photoinhibition between the two algae, nor between constant and fluctuating light treatments (Fig. 2A; see Tables S1–4 for statistical tests). Addition of lincomycin increased the light-induced decline of F_V_/F_M_ slightly in all the treatments but the difference was statistically significant only in fluctuating light in *A. acetabulum* (Fig. 2B; Tables S11–4). The results suggests that little repair occurred during the 50-min time course of the high light treatments in *A. acetabulum* and *Bryopsis* sp. (cf. Figure 2A and B). On the other hand, F_V_/F_M_ values clearly increased when the algae were let to recover for two hour after the high light treatments (without lincomycin or inhibitors) (Fig. 2C); in *A. acetabulum*, the F_V_/F_M_ values reached 89 ± 7.6% (after constant light) or 83 ± 6.2% (after fluctuating light) of the initial values but in *Bryopsis* sp., they reached only 65 ± 7.5% (after constant light) or 69 ± 7.7% (after fluctuating light) of the initial values.

**Fig. 3:**
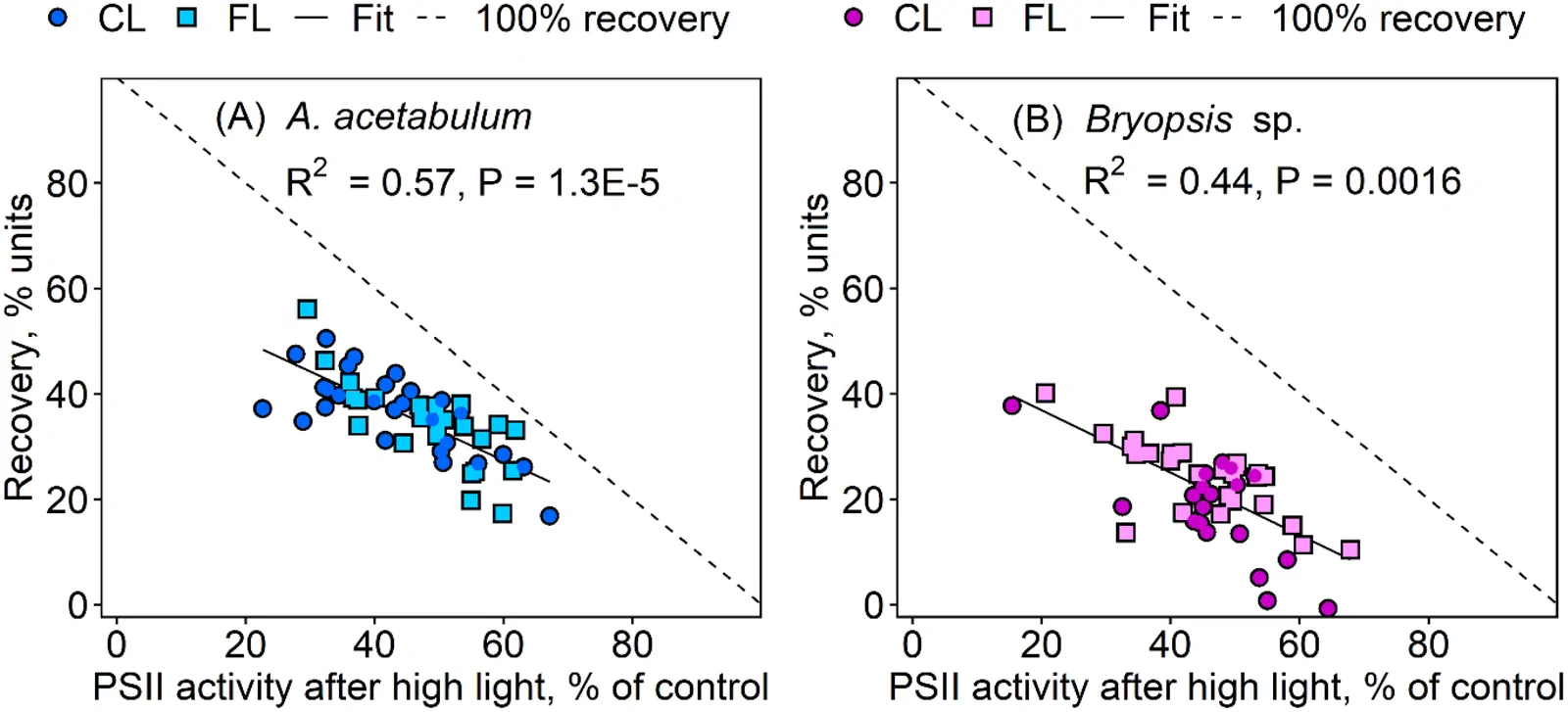
Correlations between PSII photoinhibition and recovery in *Acetabularia acetabulum* (**A**) and *Bryopsis* sp. (**B**). The algae were illuminated for 50 min with constant (circles; CL; PPFD 500 µmol m^− 2^ s^− 1^) or fluctuating (squares; FL; PPFD 0 to 1000 µmol m^− 2^ s^− 1^; see Fig. S2) white light, after which they were let to recover at low light (PPFD 10–20 µmol m^− 2^ s^− 1^) for two hours. PSII activity was probed with the chlorophyll *a* fluorescence parameter F_V_/F_M_, after 20 min in the dark; photoinhibition was quantified as F_V_/F_M_ after high light/ F_V_/F_M_ control x 100, and recovery as (F_V_/F_M_ after recovery - F_V_/F_M_ after high light)/F_V_/F_M_ control x 100. All treatments were conducted at room temperature in artificial seawater. For the original, averaged data, see Figs. 2 and S6. The solid lines show best fits to linear equations (statistics are highlighted in the corresponding panels) and the dashed lines represent (theoretical) full recovery. The symbols represent individual biological replicates

As the amount of PSII recovery was statistically significantly lower in *Bryopsis* sp. than in *A. acetabulum* (Tables S1–4), the recovery was analysed in more detail. When the amount of recovery in each individual sample (without inhibitors) was plotted against the amount of photodamage in the same sample, it was observed that the more photodamage an individual algal sample had experienced, the higher was the rate of the recovery (Fig. 3). A fit of the data (constant and fluctuating light pooled together) to a straight line was statistically significant in both algae (Fig. 3). Interestingly, even though the F_V_/F_M_ values recovered clearly less in *Bryopsis* sp. than in *A. acetabulum*, slopes of the fitted lines were very similar in *Bryopsis* sp. (-0.59) and in *A. acetabulum* (-0.56).

As NPQ relaxed slowly in *Bryopsis* sp. (Fig. 1E and F), the observed slow recovery in the F_V_/F_M_ values may not reflect slow PSII repair but instead, sustained (i.e., slowly relaxing) forms of NPQ (that is not related to photoinhibition). To obtain support for the fluorescence results, *Bryopsis* sp. was again illuminated with fluctuating light, and thylakoid membranes were isolated before and after the light treatment and after the low light recovery. PSII activity was then quantified by measuring maximum (light-saturated) oxygen evolution capacity in the presence of artificial electron acceptors from the isolated thylakoid membranes. The results (Fig. S8) support the conclusion that PSII repair occurred slowly in *Bryopsis* sp. In the oxygen evolution data, photoinhibition was observed to occur slightly slower than in the fluorescence data, which may derive from increased shading due to the high concentration of algae used (necessary to obtain enough material for thylakoid isolations) or from the relatively high variation in the control oxygen evolution activity (Fig. S8).

### Inhibitors of NPQ formation or auxiliary electron transfer pathways affected *A. acetabulum* and *Bryopsis* sp. differently

To reveal the importance of different photoprotective pathways for PSII photoinhibition and repair in *A. acetabulum* and *Bryopsis* sp., the above-described constant and fluctuating light treatments were conducted also in the presence of either antimycin A (AA; an inhibitor of the PGR5/PGRL1-mediated cyclic electron transfer route), under anoxia (to inhibit oxygen-dependent pathways, including flavodiiron activity; the oxygen concentrations remained below 25 nM during the illumination in both algae; see Fig. S9), dithiothreitol (DTT; an inhibitor of the violaxanthin to zeaxanthin conversion), nigericin (NIG; a dissipator of the proton gradient), oligomycin (OM; an inhibitor of the mitochondrial ATPase), propyl gallate (PG; an inhibitor of the PTOX) and polymyxin B (PMB; an inhibitor of the NDA2-mediated cyclic electron transfer) (Fig. 2).

In *A. acetabulum*, anoxia diminished PSII photoinhibition (i.e., the light induced decrease in the F_V_/F_M_ values) in all the treatments (constant and fluctuating high light, both in the absence and presence of lincomycin), compared to the respective treatments in plain artificial seawater under ambient air; the difference was statistically significant in all the cases, except in constant light in the presence of lincomycin (Fig. 2A and B; Tables S1 and S2). In *Bryopsis* sp., however, anoxia enhanced photoinhibition (the effect was consistent in all the treatments but statistically significant in the case of fluctuating light without lincomycin and in constant light with lincomycin; Tables S3 and S4). In *A. acetabulum*, NIG statistically significantly enhanced photoinhibition in all the treatments, compared to the treatments without NIG. On the other hand, NIG had little effects on photoinhibition in *Bryopsis* sp. PG increased photoinhibition in *Bryopsis* sp. in all the treatments (statistically significantly in the case of fluctuating light, both in the absence and presence of lincomycin) while no clear differences were found in *A. acetabulum*. In *Bryopsis* sp., DTT and OM increased photoinhibition (statistically significantly in constant light), but the effect was seen only in the absence of lincomycin (Fig. 2A and B). AA or PMB did not statistically significantly affect photoinhibition in either alga in any of the treatments.

In *A. acetabulum*, recovery under low light after the high light treatment was negatively affected by AA, DTT, NIG, PG and anoxia (Fig. 2C; Tables S1 and S2). In *Bryopsis* sp., on the other hand, only DTT statistically significantly diminished recovery (Fig. 2C; Tables S3 and S4). Interpretation of the recovery data is, however, complicated by the fact that especially AA, DTT, NIG and PG decreased PSII activity also in samples not previously treated with high light (Fig. S7) and, therefore, the observed negative effects on PSII recovery may not indicate inhibition of the repair reactions specifically.

**Fig. 4:**
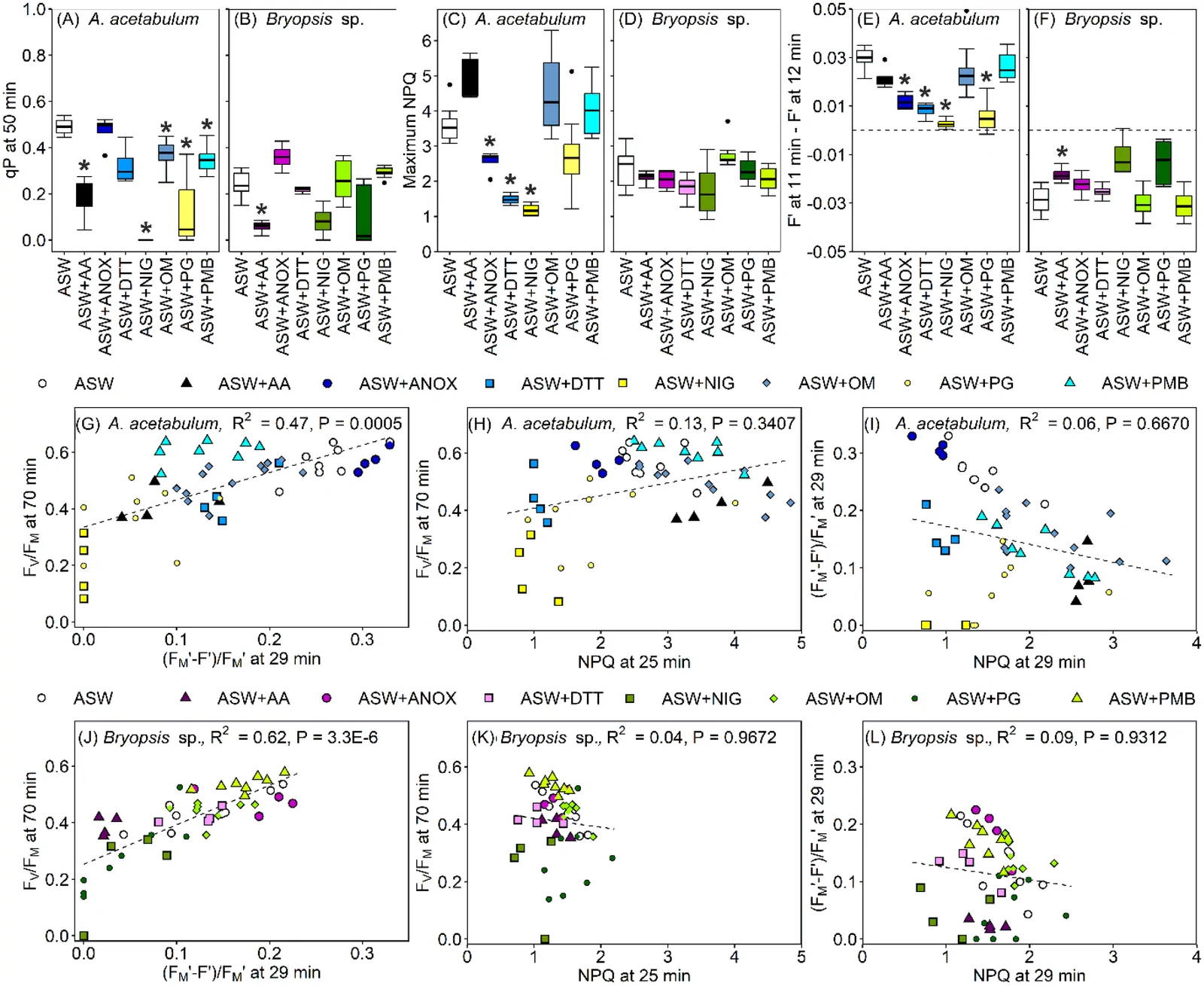
Effects of inhibitors on chlorophyll *a* fluorescence parameters in *Acetabularia acetabulum* (**A**, **C**, **E** and **G–I**) and *Bryopsis* sp. (**B**, **D**, **F** and **J–L**). Dark-acclimated algae were given a 50-min treatment with blue fluctuating light (PPFD of 0 to 1000 µmol m^− 2^ s^− 1^; Fig. S2) and subsequent 30-min dark incubation. All the treatments were conducted at room temperature in artificial seawater (ASW) supplemented either with antimycin A (AA), glucose, glucose oxidase and catalase to induce anaerobicity (ANOX; Fig. S9), dithiothreitol (DTT), nigericin (NIG), oligomycin (OM), propyl gallate (PG) or polymyxin B (PMB), as indicated. (**A** and **B**) Photochemical quenching (qP) at the indicated time points, (**C** and **D**) maximum levels of non-photochemical quenching (NPQ) during the illumination period, and (E and F) difference in incident fluorescence yield (F’) between 11 and 12 min of illumination. At 50 min, the PPFD was ~ 10 µmol m^− 2^ s^− 1^, at 11 min, ~ 120 µmol m^− 2^ s^− 1^ and at 12 min, ~ 270 µmol m^− 2^ s^− 1^. Correlations (**G** and **J**) between PSII photoinhibition (estimated as the F_V_/F_M_ values after the illumination and subsequent 20 min in the dark, i.e., after 70 min of the treatment) and PSII operational yield in light at the indicated time points, (**H** and **K**) between PSII photoinhibition and NPQ at the indicated time points, and (I and K) between PSII activity in light and NPQ. At 29 min, the PPFD was ~ 150 µmol m^− 2^ s^− 1^ and at 25 min, 1000 µmol m^− 2^ s^− 1^. The dashed lines show best fits to a linear equation (statistics are highlighted in the corresponding panels). For the original fluorescence traces, see Figs S10 and S11. The box plots (**A–F**) show medians, 2nd and 3rd quartiles, the error bars show minimum and maximum values (≤ 1.5 times the interquartile range), calculated based on four to 12 biological replicates. The asterisks highlight treatments that significantly differ from their respective controls in ASW alone (see Table S5 for details). In (**G–L**), symbols show individual biological replicates

Next, to better understand why certain inhibitors affected PSII damage and repair in *A. acetabulum* or *Bryopsis* sp., chlorophyll *a* fluorescence kinetics were re-measured under fluctuating light in plain artificial seawater as well as under anoxia or in the presence of either AA, DTT, NIG, OM, PG or PMB (Fig. 4; for the full data, see Figs S10 and S11). In both algae, majority of these chemicals negatively affected PSII operational yield (Figs S10 and S11) and consequently, qP (Fig. 4A and B). In *A. acetabulum*, the severity of the inhibition during the illumination was roughly in the order NIG > PG > AA > DTT ≈ PMB ≈ OM > anoxia (Fig. 4A); during the last high light phase of the fluctuating illumination, the differences between plain artificial seawater and the chemicals were statistically significant in the case of AA, NIG, OM, PG and PMB (Table S5). In *Bryopsis* sp., a slightly different order of effects (AA > NIG ≈ PG > DTT ≈ OM > PMB > anoxia) was observed (Fig. 4B), and only the effect of AA was statistically significant (Table S5).

As expected, DTT and NIG dramatically (and statistically significantly) decreased NPQ in *A. acetabulum* (Fig. 4C). Additionally, anoxia statistically significantly decreased NPQ in *A. acetabulum*. In *Bryopsis* sp., none of the chemicals statistically significantly affected NPQ levels (Fig. 4D).

When illuminated in plain artificial seawater, incident fluorescence yield increased in *A. acetabulum* during the low light periods of the fluctuating illumination but decreased in *Bryopsis* sp., as observed before (Fig. 1B). The increase in *A. acetabulum* greatly diminished (statistically significantly) in the treatments with DTT and NIG, supporting the idea of its relation to the relaxation of NPQ, but surprisingly, also in the PG and anaerobic treatments (Fig. 4E). In *Bryopsis* sp., only AA statistically significantly affected the behaviour of fluorescence yield during the low light periods (Fig. 4F).

To further probe how NPQ and the openness of PSII units (estimated by the PSII operational yield parameter (F_M_’-F’)/F_M_’) during illumination affected photoinhibition in *A. acetabulum* and *Bryopsis* sp., correlations between these parameters were calculated (Fig. 4G–L). In both algae, especially in *Bryopsis* sp., (F_M_’-F’)/F_M_’ after 29 min of illumination with fluctuating light (at the PPFD ~ 150 µmol m^− 2^ s^− 1^) and photoinhibition, estimated based on the F_V_/F_M_ values after 20 min in the dark (i.e. after 70 min of the experiment), showed a relatively strong correlation (Fig. 4G and J). NPQ after 25 min of illumination (at the PPFD 1000 µmol m^− 2^ s^− 1^) showed only a weak correlation with photoinhibition (Fig. 4H and K) in *A. acetabulum* and none in *Bryopsis* sp. No statistically significant correlation between (F_M_’-F’)/F_M_’ at 29 min and NPQ at the same time point was found in either of the species (Fig. 4I and L).

### High light treatment increased electron transfer in *A. acetabulum* while the opposite was observed in *Bryopsis* sp.

**Fig. 5:**
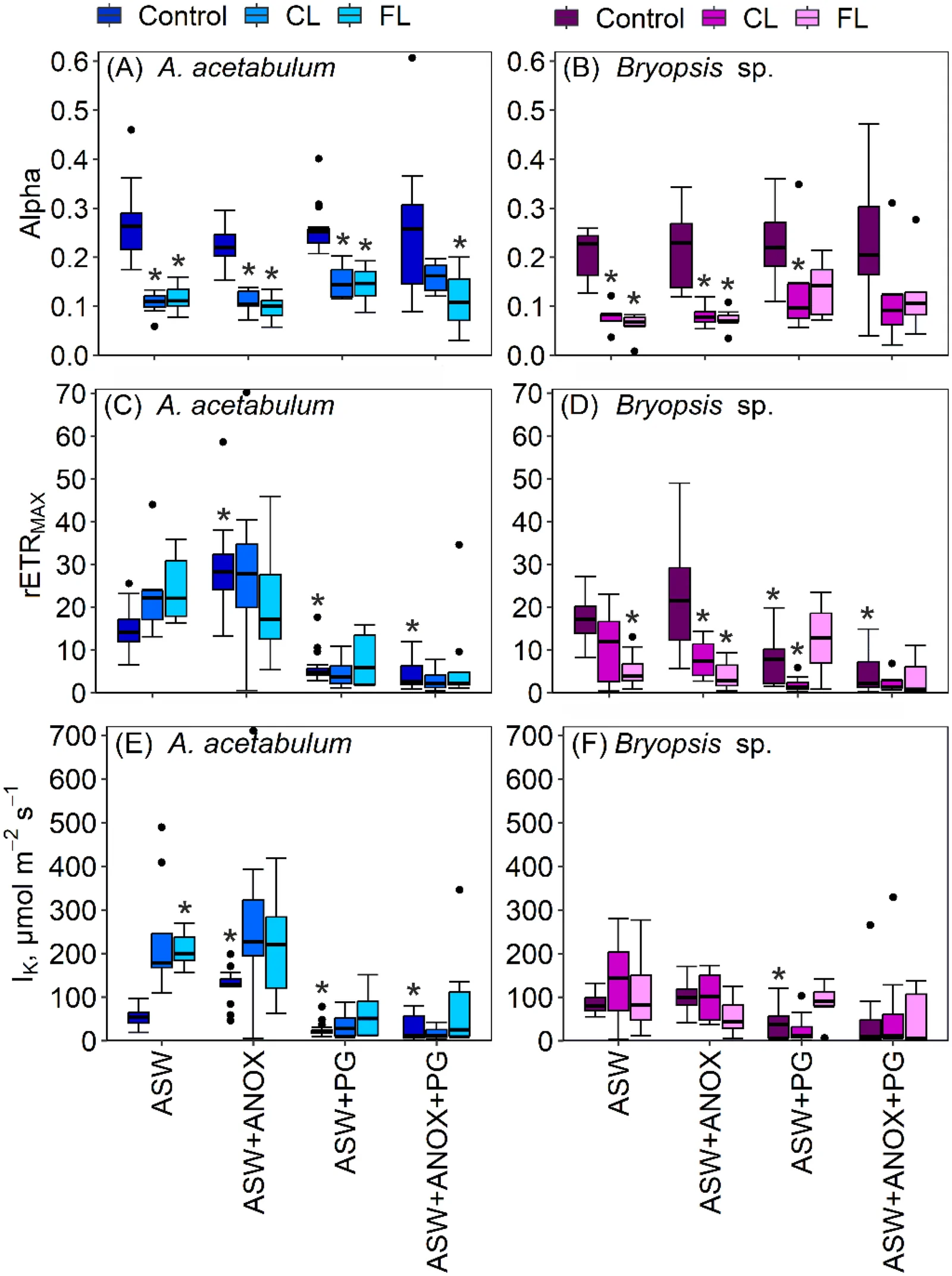
Parameters calculated based on rapid light response curves, measured with chlorophyll *a* fluorescence before (Control) and after high light treatments from *Acetabularia acetabulum* (**A**, **C**, **D**) and *Bryopsis* sp. (**B**, **D**, **F**). The algae were given a 50-min treatment with constant (CL; PPFD 500 µmol m^− 2^ s^− 1^) or fluctuating (FL; PPFD of 0 to 1000 µmol m^− 2^ s^− 1^; Fig. S2) white light. Light response curves were measured after subsequent 20-min acclimation in low light (PPFD 10–20 µmol m^− 2^ s^− 1^). During the light response curves, the algae were illuminated with increasing intensities of blue light (for 60 s at each intensity); saturating light pulses were fired to calculate relative rates of electron transfer. All measurements were conducted at room temperature in artificial seawater (ASW), supplemented with the following chemicals (added 20 min prior to the measurements): glucose, glucose oxidase and catalase (ANOX; to induce anaerobicity; Fig. S9), propyl gallate (PG) or both, as indicated. To calculate alpha (initial slope of the light response curve; **A–B**), maximum relative electron transfer rate (rETR_MAX_; **C–D**) and minimum saturating intensities (I_K_; **E–F**), the curves were fitted to the model of Eilers and Peeters (1988). See Fig. S12 for the original fluorescence curves and examples of the fits. The box plots show medians, 2nd and 3rd quartiles, error bars show minimum and maximum values and dots show outliers (> 1.5 times the interquartile range), calculated based on eight to 16 biological replicates. The asterisks highlight treatments that statistically significantly differ from their respective controls, or in the case of control, from the chemical-treated controls (see Tables S6 and S7 for details)

As the PSII operational yield under high light had a stronger relationship with the amount of PSII photoinhibition in *Bryopsis* sp. than in *A. acetabulum* (Fig. 4), electron transfer rates were next probed by measuring rapid light response curves with chlorophyll *a* fluorescence (Fig. S12) before and after the constant and fluctuating light treatments. To obtain alpha (the initial slope of the rapid light response curve), rETR_MAX_ (the maximum rate of the relative electron transfer) and I_K_ (the minimum saturating irradiance) values, the light response curves were fitted to the model of Eilers and Peeters (1988). Unsurprisingly, the alpha parameter decreased after the high light treatments in both algae (Fig. 5A and B). However, rETR_MAX_ and I_K_ increased in *A. acetabulum* (though the increase was statistically significant only in the case of I_K_ after fluctuating light; Fig. 5, Table S6). In *Bryopsis* sp., on the other hand, rETR_MAX_ slightly decreased after the high light treatments (statistically significantly after fluctuating light; Fig. 5, Table S7).

Anoxia and PG had opposite effects on photoinhibition in *A. acetabulum* and *Bryopsis* sp. (Fig. 2). Therefore, their effects on rapid light response curves were measured, as well as their combined effect (Fig. 5). Anoxia increased rETR_MAX_ and I_K_ values in non-illuminated *A. acetabulum* whereas no further increases were observed in the high light-treated samples (Fig. 5C and E). No significant effects were observed in *Bryopsis* sp. (Fig. 5D and F). PG as well as the combined treatment with anoxia and PG clearly diminished electron transfer rates in both algae (Fig. 5C–F).

**Fig. 6:**
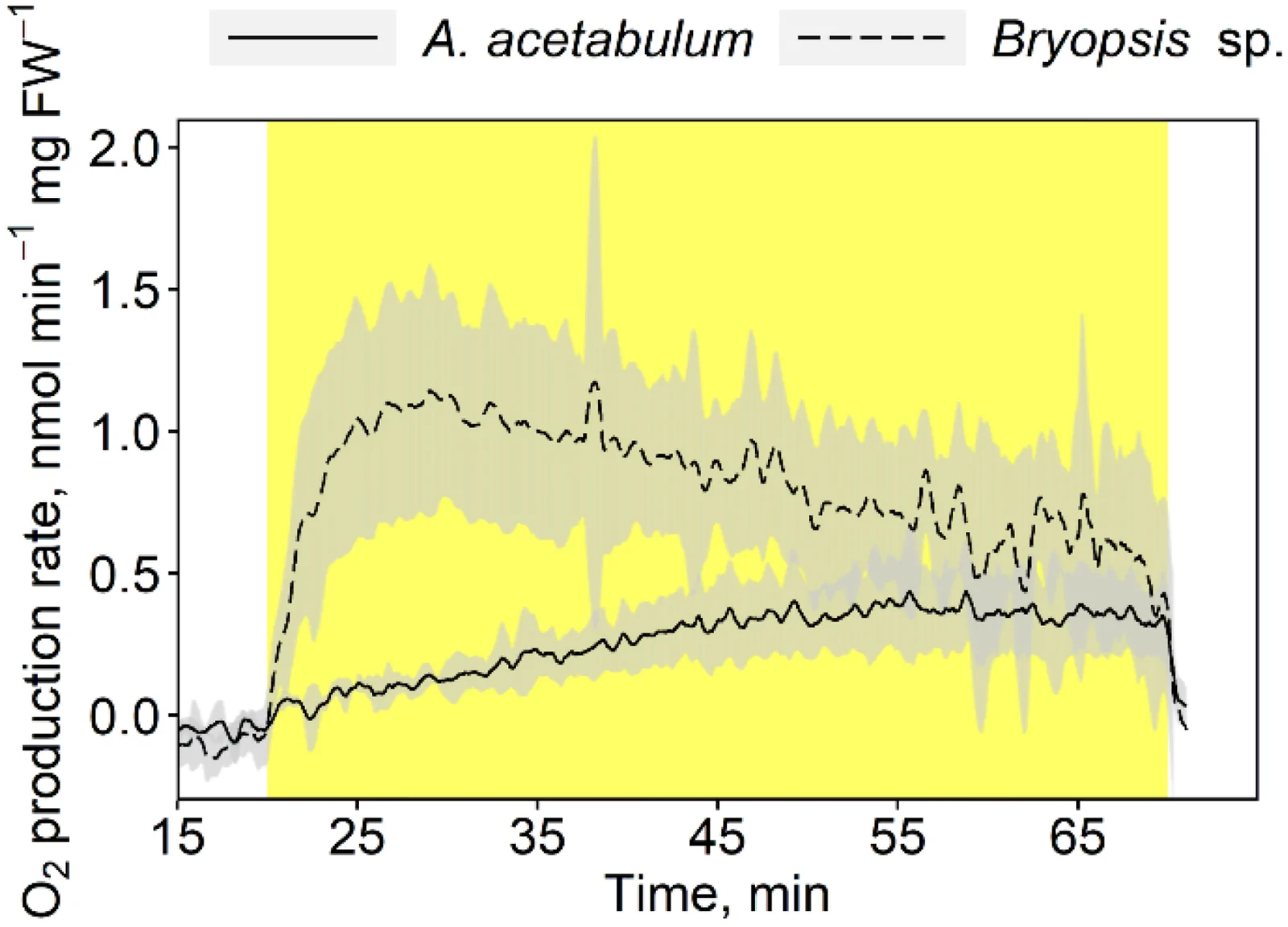
Net oxygen consumption (in the dark) or evolution (in the light) by intact cells of *Acetabularia acetabulum* and *Bryopsis* sp. during a 20-min darkness and subsequent 50-min illumination with constant white light (illustrated with the yellow panel; PPFD 500 µmol m^− 2^ s^− 1^) at 20 °C in artificial seawater. The oxygen production/consumption has been calculated on a fresh weight (FW) basis of the sample. The lines show averages and the shaded areas standard deviations calculated based on five to six biological replicates

As fluorescence-based estimations of electron transfer rate rely on assumptions that were not verified in the present study (i.e., whether the PSII absorption cross sections were similar in the two algae is not known), the rETR_MAX_ values cannot be directly compared between *Bryopsis* sp. and *A. acetabulum*. Thus, we next measured the rates of net oxygen evolution (or consumption) from intact cells (Fig. 6). In *A. acetabulum*, in accordance with the rETR_MAX_ measurements, the rate of oxygen evolution increased during the illumination, up to ~ 25 min under the treatment (constant white light), whereas *in Bryopsis* sp., high rates of oxygen evolution were observed almost immediately after switching on the light, after which the rate steadily decreased (Fig. 6).

**Fig. 7:**
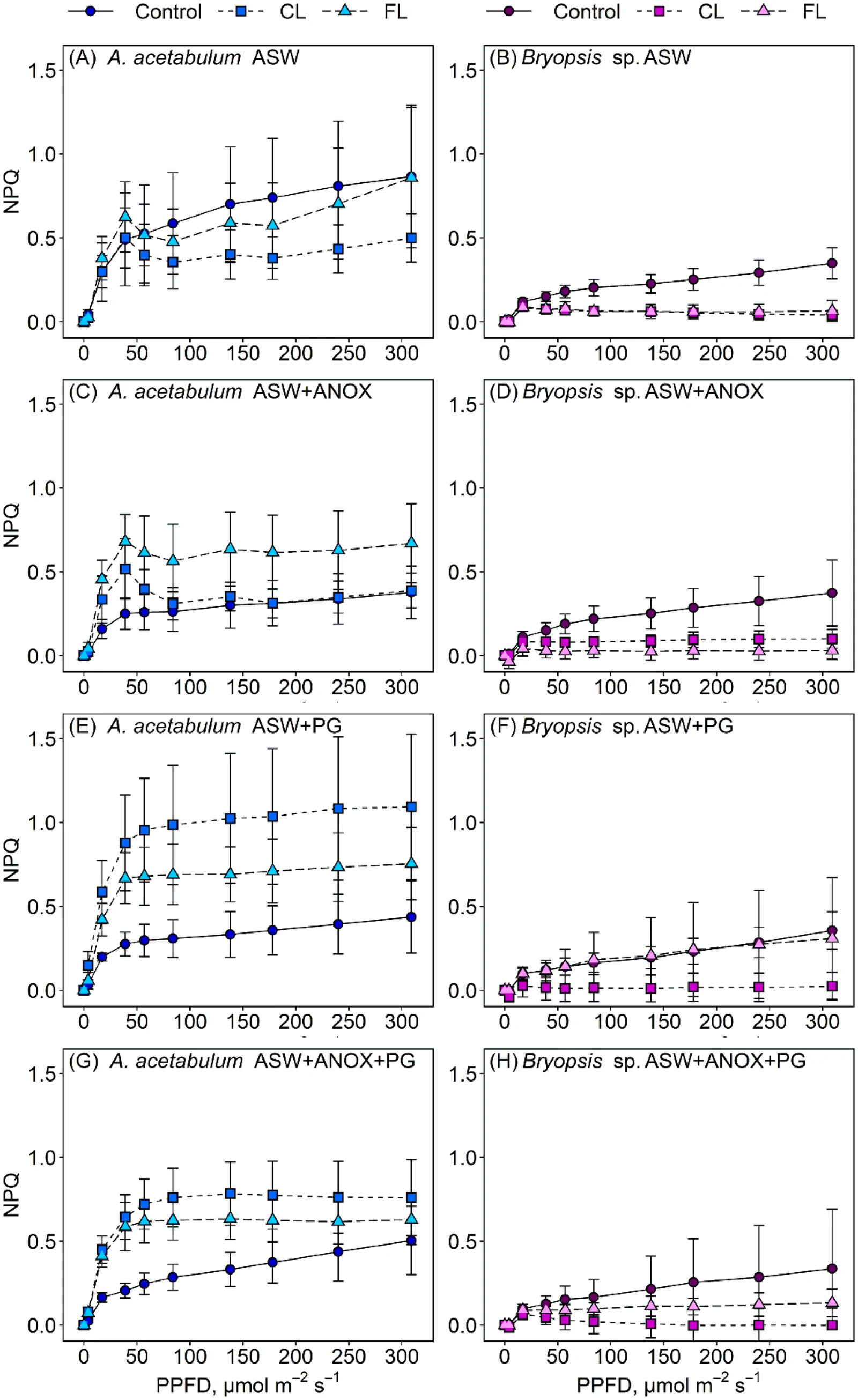
Non-photochemical quenching (NPQ), measured with chlorophyll *a* fluorescence, during rapid light response curves before (Control) and after high light (constant or fluctuating) treatments in *Acetabularia acetabulum* (**A**, **C**, **E**, **G**) and *Bryopsis* sp. (**B**, **D**, **F**, **H**). The algae were given a 50-min treatment with constant (CL; PPFD 500 µmol m^− 2^ s^− 1^) or fluctuating (FL; PPFD of 0 to 1000 µmol m^− 2^ s^− 1^; Fig. S2) white light. Light response curves were measured after subsequent 20 min acclimation in low light (PPFD of 10–20 µmol m^− 2^ s^− 1^). During the light response curves, the algae were illuminated for 60 s with increasing intensities of blue light, as indicated; after each intensity, a saturating light pulse was fired to calculate NPQ. All the treatments were conducted at room temperature in artificial seawater (ASW) (**A** and **B**), supplemented with the following chemicals (added 20 min prior to the measurements): glucose, glucose oxidase and catalase (ANOX; to induce anaerobicity; Fig S9; **C** and **D**), propyl gallate (PG; **G** and **F**) or both (**E** and **F**), as indicated. The symbols show averages and error bars standard deviations calculated based on eight to 16 biological replicates

NPQ formed during the light response curves was also quantified; in general, NPQ induction decreased after the high light treatments, especially in *Bryopsis* sp. (Fig. 7). However, the used 20 min low light acclimation prior to the measurement may not have been enough to relax all NPQ, especially in the case of *Bryopsis* sp., which may have affected the results. In *A. acetabulum*, a transient peak in NPQ appeared after the high light treatments, which did not form in the presence of PG (Fig. 7E and G). In the PG-treated *A. acetabulum* samples, high light-exposed algae induced more NPQ than in the absence of PG, which may be related to the slow rates of electron transfer in these samples. In accordance with the previous fluorescence measurements (Fig. 4D), the chemicals had little effect on NPQ in *Bryopsis* sp. (Fig. 7).

### Inhibitors of cyclic electron transfer had a bigger effect in *A. acetabulum* than in *Bryopsis* sp.

**Fig. 8:**
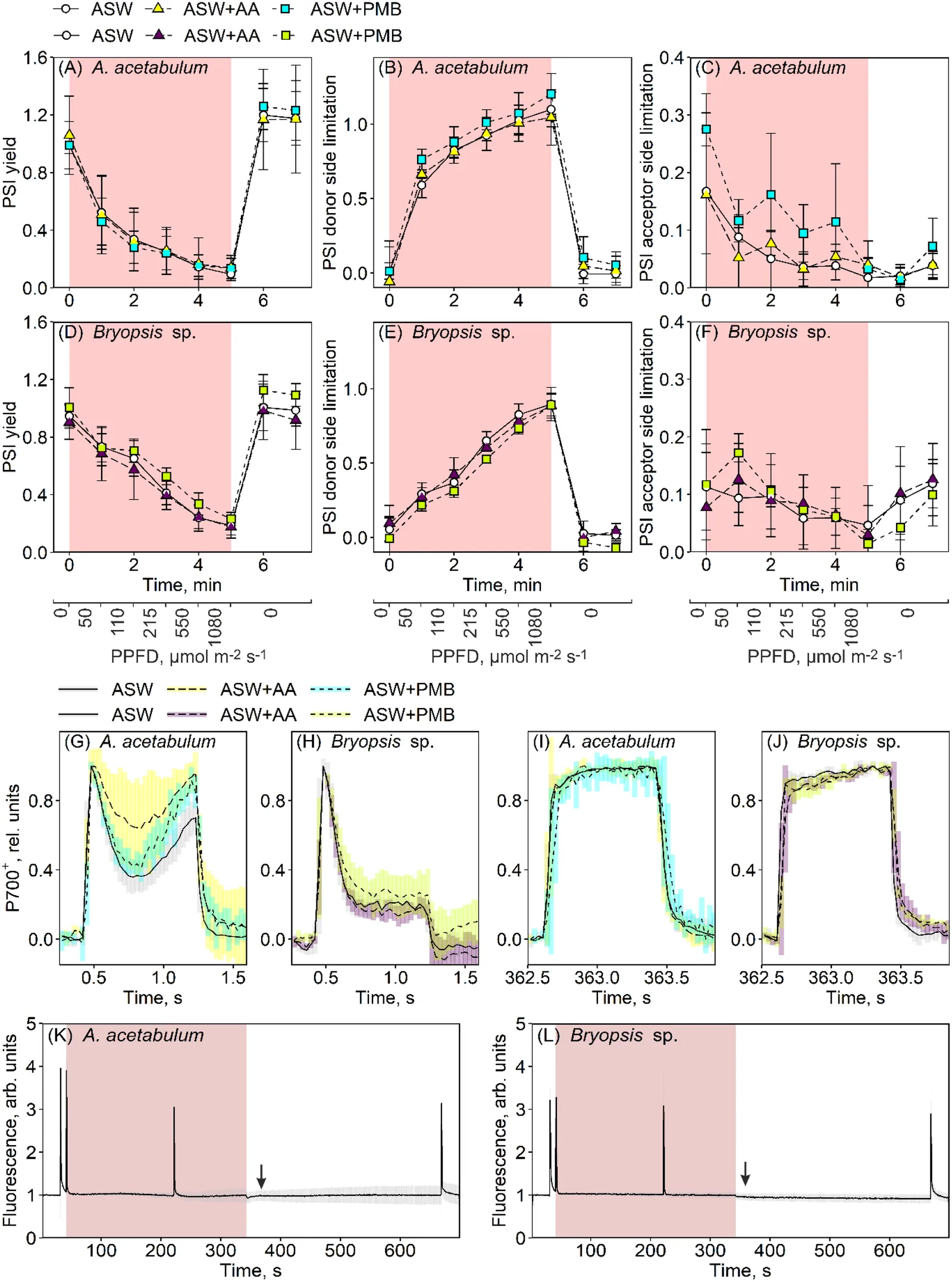
Photosystem I (PSI) kinetics and cyclic electron transfer in *Acetabularia acetabulum* (**A–C**, **G**, **I** and **K**) and *Bryopsis* sp. (**E–F**, **H**, **J** and **L**). P700 oxidation was measured from dark-acclimated algae under increasing intensities of red light (PPFDs 50, 110, 215, 550 and 1080 µmol m^− 2^ s^− 1^; one min with each intensity; illustrated with the light red panel) and during the subsequent two-min darkness, in the absence or presence of either antimycin A (AA) or polymyxin B (PMB), as indicated. Saturating light pulses were fired to calculate (**A** and **D**) PSI yield, (**B** and **E**) donor side limitation and (**C** and **F**) acceptor side limitation. (**G** and **H**) P700 kinetics during the first saturating light pulse (from dark acclimated algae) and (**I** and **J**) during the first saturating light pulse after switching off the red-light illumination. The P700^+^ signals are normalized between minimum and maximum values, to facilitate comparison. See Fig. S13 for examples of the full data. (**K** and **L**) Chlorophyll *a* fluorescence kinetics during far-red light (PFD 50 µmol m^− 2^ s^− 1^; illustrated with the red panel) and subsequent darkness. The arrows highlight the post-illumination F_O_ rise (or its absence). All treatments were conducted at room temperature. Symbols show averages and error bars standard deviations calculated based on three to five biological replications

The inhibitors of PSI cyclic electron transfer (AA and PMB) did not have a big effect in PSII photoinhibition and recovery in either of the algae (Fig. 2). Therefore, their effects on the redox state of PSI were assayed by measuring P700 oxidation by near-infra-red absorption (Figs. 8A–J and S13). When illuminated with increasing intensities of red light (PPFD ~ 50–1000 µmol m^− 2^ s^− 1^), PSI yield decreased, faster in *A. acetabulum* than in *Bryopsis* sp., whereas donor side limitation increased and acceptor side limitation decreased in both algae (Fig. 8A–F). In *Bryopsis* sp., donor side limitation was clearly lower than in *A. acetabulum*, especially under low light (PPFD ~ 50–200 µmol m^− 2^ s^− 1^). However, the presence of AA or PMB had little effect on any of the parameters; PMB slightly increased acceptor side limitation in *A. acetabulum* (Fig. 8C). In the case of dark-acclimated algae, P700 oxidation during saturating light pulses rapidly increased, then decreased and, in the case of *A. acetabulum* but not in *Bryopsis* sp., again increased (Fig. 8G and H). In contrast, after the illumination treatment, P700 stayed oxidized during the whole 800-ms duration of the saturating light pulse in both algae (Fig. 8I and J). In *Bryopsis* sp., AA and PMB had no effect on the P700 oxidation kinetics, but in *A. acetabulum*, the initial peak in P700 oxidation was less pronounced in the presence of both chemicals, especially in the case of AA.

Finally, the amount of cyclic electron transfer in *A. acetabulum* and *Bryopsis* sp. was estimated by measuring the chlorophyll *a* fluorescence post illumination F_O_ rise (Fig. 8K and L); *A. acetabulum* showed a small F_O_ rise, but no rise was observed in *Bryopsis* sp.

## Discussion

Bryopsidales algae seem to miss the fast, energy-dependent component of NPQ (qE) (Christa et al. 2017) as well as state transitions (Havurinne et al. 2025), two photoprotective mechanisms previously shown to be especially important under conditions of high and fluctuating light (e.g., Cantrell and Peers 2017; Steen et al. 2022). Here, we show that in the Bryopsidales alga *Bryopsis* sp., PSII photoinhibition, probed by the chlorophyll *a* fluorescence parameter F_V_/F_M_, did not occur faster under fluctuating light than under constant light (Fig. 2). The same occurred in *A. acetabulum*, an Ulvophyceaen green macroalga with similar siphonous morphology and light absorptance to *Bryopsis* sp. (Figs S1 and S3) but clear capacities for both qE (Fig. 1) and state transitions (Havurinne et al. 2025). The high light-induced decline in the F_V_/F_M_ values also proceeded at similar rates in the two algae, suggesting similar rates of photodamage; however, the different fluorescence kinetics of the algae may have affected the results. Regardless, even if the damaging reaction of photoinhibition did not proceed especially fast in *Bryopsis* sp., the alga seemed to repair the PSII damage more slowly than *A. acetabulum* (Figs. 2 and 3), and in the alga, high light treatments also led to a decrease in electron transfer rates, which was not observed in *A. acetabulum* (Figs. 5 and 6).

### *A. acetabulum* relies on NPQ for photoprotection while *Bryopsis* sp. might utilize oxygen-dependent pathways

Upon high light (constant or fluctuating) treatments, *Bryopsis* sp. slowly induced strong NPQ that also relaxed slowly (Fig. 1); a strong NPQ has been previously suggested to contribute to photoprotection in *Bryopsis corticulans* (Giovagnetti et al. 2018). Here, *A. acetabulum*, too, induced similar, high levels of NPQ, but as the induction occurred rapidly in *A. acetabulum*, the difference in NPQ levels between *Bryopsis* sp. and *A. acetabulum* was large during the first ~ 10 min of illumination. Compared to *A. acetabulum*,* Bryopsis* sp. also showed less PSI donor side limitation (Fig. 8) (possibly due to a weak photosynthetic control, as decreased NPQ levels did not affect PSI donor side limitation in plant mutants deficient in qE; see e.g., Barbato et al. 2020). Therefore, some damage might be expected to occur in *Bryopsis* sp. especially during the first minutes of high light illumination. However, NPQ levels did not correlate well with PSII photoinhibition in either of the species (Fig. 4). In the case of *A. acetabulum*, the ability to switch NPQ rapidly on may be more important than the actual NPQ levels, as removal of the proton gradient with NIG (i.e., removal of the capacity for fast NPQ induction/relaxation) increased the rate of the photodamage to PSII while inhibition of the xanthophyll cycle with DTT (causing a clear decrease in maximum NPQ while some capacity for fast induction/relaxation remained; Fig. 4) did not (Fig. 2).

In *A. acetabulum*, anoxic (or microoxic; Fig. S9) conditions diminished PSII damage, an effect previously observed in plant leaves (Fan et al. 2016), possibly due to decreased production of singlet oxygen (Hideg et al. 1994; Pfleger et al. 2024). Interestingly, the opposite was true for *Bryopsis* sp. (Fig. 2), suggesting that oxygen-dependent pathways (flavodiiron proteins, PTOX and/or mitochondrial respiration) are more important for *Bryopsis* sp. than for *A. acetabulum*. High rates of photoinhibition of both photosystems previously observed in *B. corticulans* chloroplasts under anaerobic conditions were also concluded to be related to electron transfer through PSI (Satoh and Fork 1982). The importance of PTOX and, perhaps to a lesser degree, mitochondrial respiration for *Bryopsis* sp. is supported by the increased PSII damage in the presence of PG and OM (Fig. 2), while in *A. acetabulum*, these chemicals did not significantly affect photoinhibition. *Bryopsis* sp. inhabits intertidal coastal waters that are often rich in oxygen during daytime (e.g., Truchot and Duhamel-Jouve 1980); under these conditions, the oxygen-dependent auxiliary pathways may offer feasible photoprotective mechanisms.

Microoxic conditions did not decrease the electron transfer rates (estimated by chlorophyll *a* fluorescence) in either of the algae (Fig. 5), nor did they lower photochemical quenching (qP) under fluctuating light (Fig. 4), suggesting that oxygen-dependent pathways did not significantly contribute to the linear electron transport rates, but rather work transiently upon sudden increases or decreases in light intensity. Transient activation of flavodiiron-dependent oxygen consumption has been previously observed during dark to light transitions (Santana-Sanchez et al. 2019; Saroussi et al. 2019). Here, in dark-acclimated Bryopsis sp. cells, P700 was only transiently oxidised (during a high light pulse), in contrast to *A. acetabulum* and other photosynthetic organisms possessing flavodiiron-proteins, where P700 usually stays oxidized during the whole light pulse (Fig. 8; see e.g., Shimakawa et al. 2019). Previously, the Bryopsidales alga *Codium fragile* has been shown to possess clear flavodiiron activity (Shimakawa et al. 2019). Indeed, after a short illumination, P700 stayed oxidized in both algae, suggesting that in *Bryopsis* sp., flavodiiron activity might be downregulated under darkness more strongly than in *A. acetabulum*. Of course, activities of the cyclic electron transfer pathways (discussed below) may also affect P700 oxidation. On the other hand, PG strongly suppressed PSII electron transfer (Figs. 4 and 5). However, the result may not indicate high PTOX activity, as removal of oxygen (where no decrease in electron transfer was observed) should also hinder PTOX, but may be a side effect of PG.

PSII photoinhibition most likely proceeds via multiple pathways, some of which are oxygen-dependent and others oxygen-independent (Mattila et al. 2022a). Possibly, the oxygen-dependent routes (related to singlet oxygen) are less important than the oxygen-independent routes (light absorption by the oxygen evolving complex and/or oxidation by P680^+^) in causing PSII damage in *Bryopsis* sp. compared to *A. acetabulum*. Alternatively, increased photoinhibition under microoxic conditions could derive from increased superoxide production by PSI, due to inactivation of the flavodiiron activity (Song et al. 2006; Pfleger et al. 2024). However, often only the ROS produced by PSII itself (mostly singlet oxygen) are considered to be relevant for PSII damage, due to strong antioxidative capacities of cells (Herbert et al. 1992; Nishiyama et al. 2001; Tyystjärvi 2013; Sae-Tang et al. 2016).

PSII operational yield correlated with PSII photoinhibition more strongly in *Bryopsis* sp. than in *A. acetabulum* (Fig. 4). Accordingly, the high light treatments lowered electron transfer rates and net oxygen evolution in *Bryopsis* sp., while in *A. acetabulum* these parameters increased after illumination (Figs. 5 and 6). However, the data suggest that fast photoinhibition lowers electron transfer rates in *Bryopsis* sp. (rather than the low electron transfer rates causing photoinhibition) as during the early time points of the fluctuating illumination, i.e., after 1 and 9 min of illumination, weaker correlations (R^2^ = 0.48 and R^2^ = 0.51, respectively) were found between the above-mentioned parameters than in the later time points, under the same light intensities (R^2^ = 0.62; Fig. 4). Furthermore, NIG, which also strongly suppressed PSII operational yield as well as photochemical quenching (Fig. 4), did not significantly increase photoinhibition in *Bryopsis* sp. (Fig. 2).

### Why does *Bryopsis* sp. repair damaged PSII units slowly?

Compared to *A. acetabulum*, *Bryopsis* sp. showed low rates of PSII recovery (Figs. 2 and 3 and S8). The slightly lower control (prior to any high light stress) F_V_/F_M_ values of *Bryopsis* sp. (0.70 ± 0.03) compared to *A. acetabulum* (0.73 ± 0.03) could also indicate sustained photoinhibition due to an incomplete PSII repair. The synthesis of the new D1-protein during the PSII repair cycle is known to be sensitive to ROS, due to oxidation of the elongation factors EF-G and EF-Tu (Kojima et al. 2007; Yutthanasirikul et al. 2016). In *A. acetabulum*, the strongly suppressed PSII recovery under microoxic conditions (Fig. 2) could be explained by increased superoxide production by PSI (Pfleger et al. 2024). In contrast, PSII recovery was not affected by anoxia in *Bryopsis* sp. (Fig. 2). Furthermore, in both algae, recovery rates increased with increasing rates of damage (Fig. 3), suggesting that the slow PSII recovery in *Bryopsis* sp. was not due to decreased repair capacity (caused by, e.g., excess ROS production), but rather due to downregulation of the repair activity. Future studies should, perhaps, confirm the results using other methods (e.g., oxygen evolution or PSI kinetics), to exclude the possibility that different rates of NPQ relaxation in different samples interfered with the chlorophyll *a* fluorescence-based estimations of PSII repair rates.

Why didn’t the algae, especially *Bryopsis* sp., repair all the damage? PSII repair requires energy (Murata and Nishiyama 2018; Yi et al. 2022) and decreased rates of protein synthesis (including the PSII reaction centre protein D1) under oxidative stress has been suggested to be a regulatory response, to prevent excessive ATP consumption, but also to prevent PSI damage and/or further ROS production (Tikkanen et al. 2014; Yutthanasirikul et al. 2016; Murata and Nishiyama 2018; Cheng et al. 2024). In plants, moderate photoinhibition does not decrease carbon fixation rates under high light, suggesting that plants contain “extra” PSII units (Mattila et al. 2020). Also in *A. acetabulum*, high light treatments rather increased PSII electron transfer rate (Figs. 5 and 6) and thus, sustained photoinhibition may not prevent efficient photosynthesis in this alga. Therefore, it could be beneficial to wait for the stress to pass, before repairing all the damage. This may be especially true for environments, such as tidal pools, where high light stress typically does not last for long. However, the opposite was true for *Bryopsis* sp., where electron transfer rates decreased during and after high light illumination (Figs. 5 and 6), suggesting that the alga loses opportunities for carbon fixation due to the accumulation of non-functional PSII units. Relatively high F_V_/F_M_ values (> 0.55) during low tide or at noon are reported for Bryopsidales algae growing in the field (Raniello et al. 2006; Giovagnetti et al. 2018), suggesting ongoing PSII repair. In addition, no decrease in electron transfer rates at noon was reported in the field-grown alga (Raniello et al. 2006), suggesting that photosynthetic reactions are differentially regulated when the algae are grown under low light (here) and under high light (in the field). Accordingly, regulation of photosynthesis was found to be one of the upregulated gene ontology categories in high light treated *B. corticulans* (Xu et al. 2025). It can be speculated that *Bryopsis* sp. upregulates PSII repair when grown under high light, as occurs in plants (e.g., Miyata et al. 2015), to avoid losing changes for carbon fixation. However, little is known about the regulation of PSII repair in (macro)algae.

ATP deficiency due to decreased mitochondrial respiration has been previously suggested to hinder repair in seagrasses under anoxia (Che et al. 2023), which could also explain the low rates of PSII recovery under microoxic conditions in *A. acetabulum* (Fig. 2). However, *A. acetabulum* showed high electron transfer rates under anoxia (Fig. 5), suggesting no ATP deficiency. In addition, inhibition of the mitochondrial respiration did not affect repair in either of the algae (Fig. 2). In plants, PSI cyclic electron transfer is important for efficient PSII repair (Huang et al. 2018). Here, inhibition of the cyclic electron transfer did not greatly affect PSII repair in either of the algae; AA did slow down repair in *A. acetabulum*, but the effect could be due to inactivation of PSII by AA under darkness (Fig. S7). In *Bryopsis* sp., neither of the inhibitors (AA or PMB) affected PSI oxidation and no F_O_ rise (a chlorophyll *a* fluorescence method used to estimate PSI cyclic electron transfer) was observed, unlike in *A. acetabulum* (Fig. 8). The results may suggest that the low-light-grown *Bryopsis* sp. possess low capacity for cyclic electron transfer. Alternatively, it is possible that the used inhibitors did not work in the Bryopsidales alga. However, cross-compensation among the different auxiliary electron pathways (Chaux et al. 2017b; Peltier et al. 2024) may also explain the lack of effect of a single inhibitor.

The increased electron transfer rate (probed by chlorophyll *a* fluorescence) in *A. acetabulum* under anoxia (Fig. 5) is curious. As also high light treatment increased electron transfer rates in this alga (Figs. 5 and 6), it seems plausible that electron transfer in *A. acetabulum* is downregulated under low light and anoxia, perhaps, disturbs this regulation. Alternatively, hydrogenase activity, previously shown to be important under anaerobic conditions in *C. reinhardtii* (Godaux et al. 2015), may increase under anoxia.

## Concluding remarks

Compared to the Dasycladales alga *A. acetabulum*, the Bryopsidales alga *Bryopsis* sp. responds to high light treatments very differently, as probed by measuring pigment compositions, chlorophyll *a* fluorescence, oxygen evolution and P700 kinetics. Despite the lack of rapid NPQ induction, PSII of *Bryopsis* sp. appears to be well protected against high light. Previously, the protective effect of NPQ against PSII damage has been shown to be rather small (for a review, see Tyystjärvi 2013), which agrees with the present results. Regardless, in the absence of qE, oxygen-dependent pathways seemed to be especially important for *Bryopsis* sp.. Future functional and protein level studies should verify these results, as the current conclusion about the importance of the oxygen-dependent pathways rely mainly on data obtained with chemical inhibitors, which may have undesired side-effects. In addition, this study describes short treatments done on low-light-grown algae; high light acclimated algae may rely on different photoprotective mechanisms.

Finally, it should be noted that both *Bryopsis* sp. and *A. acetabulum* are capable of fast cytoplasmic streaming; extensive chloroplast movements could thus disturb the present chlorophyll *a* fluorescence-based measurements (especially NPQ). Recently, we attempted to quantify the movements; in *A. acetabulum*, chloroplast movements respond to high light and appear to function as a photoprotective mechanism, but their magnitude is limited, particularly in *Bryopsis* sp. (Mattila et al. 2026). Thus, the potential contributions of chloroplast movement to the present chlorophyll *a* fluorescence results are expected to be minor.

## Supplementary Information

Below is the link to the electronic supplementary material.

Supplementary Material 1

## Data Availability

The raw data will be available upon publication at Mendeley Data (doi: 10.17632/j2xpnzyk3c.1).
