# Supplementary material for: Siphonous green macroalgae with contrasting capacities for the energy-dependent quenching, qE, rely on different photoprotective mechanisms
Source: Photosynth Res. 2026 Jun 26;164(4):37. doi: 10.1007/s11120-026-01225-1 (PMC13309506; doi:10.1007/s11120-026-01225-1)
Supplement: Supplementary file 1 — Supplementary Material 1 [file 11120_2026_1225_MOESM1_ESM.docx]

Supplementary information for:

**Siphonous green macroalgae with contrasting capacities for the energy-dependent quenching, qE, rely on different photoprotective mechanisms**

**Heta Mattila^1,2,#^, Vesa Havurinne^1^,** **Paulo Cartaxana^1^, Sónia Cruz^1^**

^1^CESAM–Centre for Environmental and Marine Studies, Department of Biology, University of Aveiro, Portugal; ^2^Molecular Plant Biology, Department of Life Technologies, University of Turku, Finland; ^#^Corresponding author email: [hkmatt@utu.fi](mailto:hkmatt@utu.fi)

**Supplementary figures**


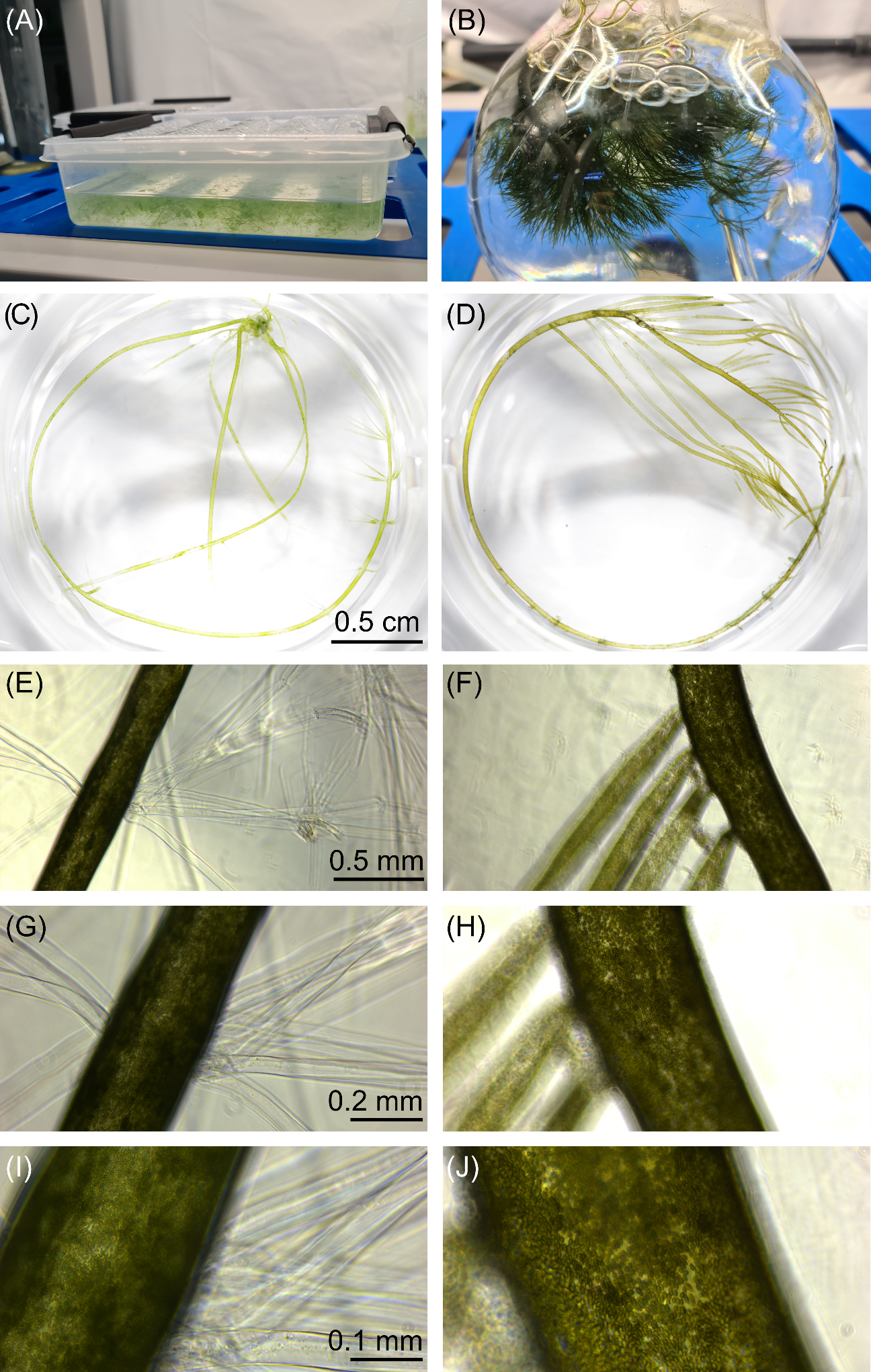


**Fig. S1**. Exemplary images of *Acetabularia acetabulum* (A, C, E, G and I) and *Bryopsis* sp. (B, D, F, H, J). (A and B) Culture conditions. (C and D) Macro-photographs of whole algal cells, placed in a plastic 6x4 well plate. (E–J) Light microscopy images of a part of an algal cell, with 5x (E and F), 10x (G and H) and 20x (I and J) objectives. The image pairs (C and D), (E and F), (G and H) and (I and J) have the same scales and the scale bar is shown in the left image.

**
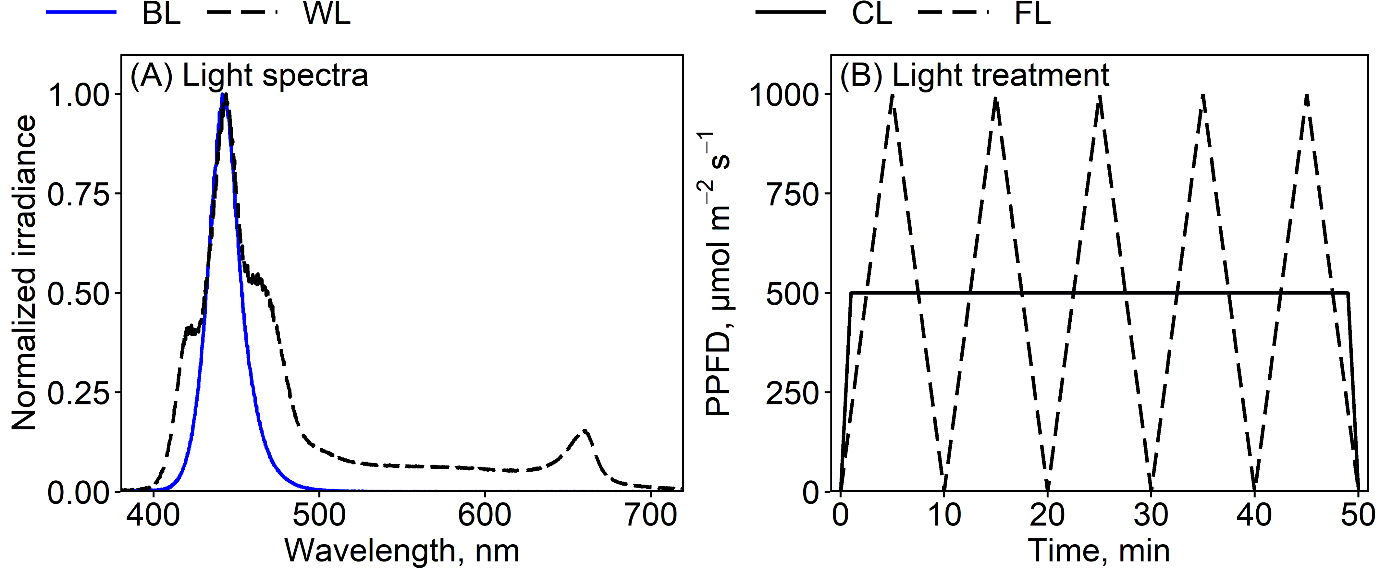
**

**Fig. S2**. Light treatments. (A) Normalized energy spectra of the blue (BL; Imaging-PAM, Walz, Germany) and white (WL; Reef Pulsar SPS-8, Tropical Marine Centre, UK) light sources used in the high light treatments in the present study. (B) A schematic presentation of the constant (CL) and fluctuating (FL) light treatments, used in the present study. The cumulative amount of light was the same in both the treatments. PPFD = photosynthetic (400–700 nm) photon flux density.


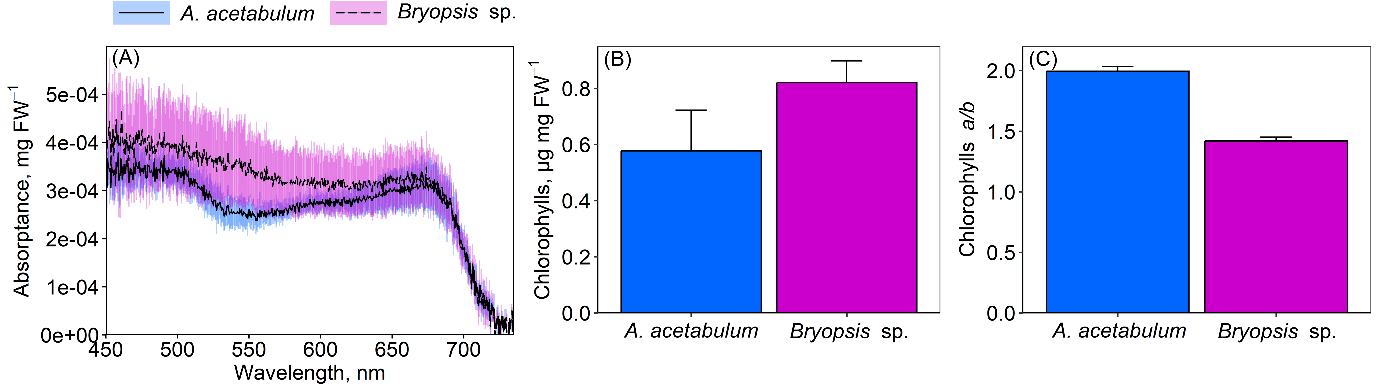


**Fig. S3**. Absorptances, chlorophyll contents and chlorophyll *a/b* ratios of *Acetabularia acetabulum* and *Bryopsis* sp.. (A) Absorptances of intact algae, measured in an integrating sphere and normalized to fresh weight (FW), in *A. acetabulum* (continuous lines with blue shading) and *Bryopsis* sp. (dashed lines with purple shading). Absolute values of light absorption were not calculated and background signals at 750 nm have been subtracted from the final values. (B) Contents of chlorophylls *a* + *b*, per fresh weight, and (C) ratios of chlorophylls *a* to *b*, measured spectrophotometrically after extraction in dimethylformamide, in *A. acetabulum* (blue bars) and *Bryopsis* sp. (purple bars). Lines and bars show averages and shaded areas (A) or error bars (B and C) standard deviations, calculated based on three biological replicates.


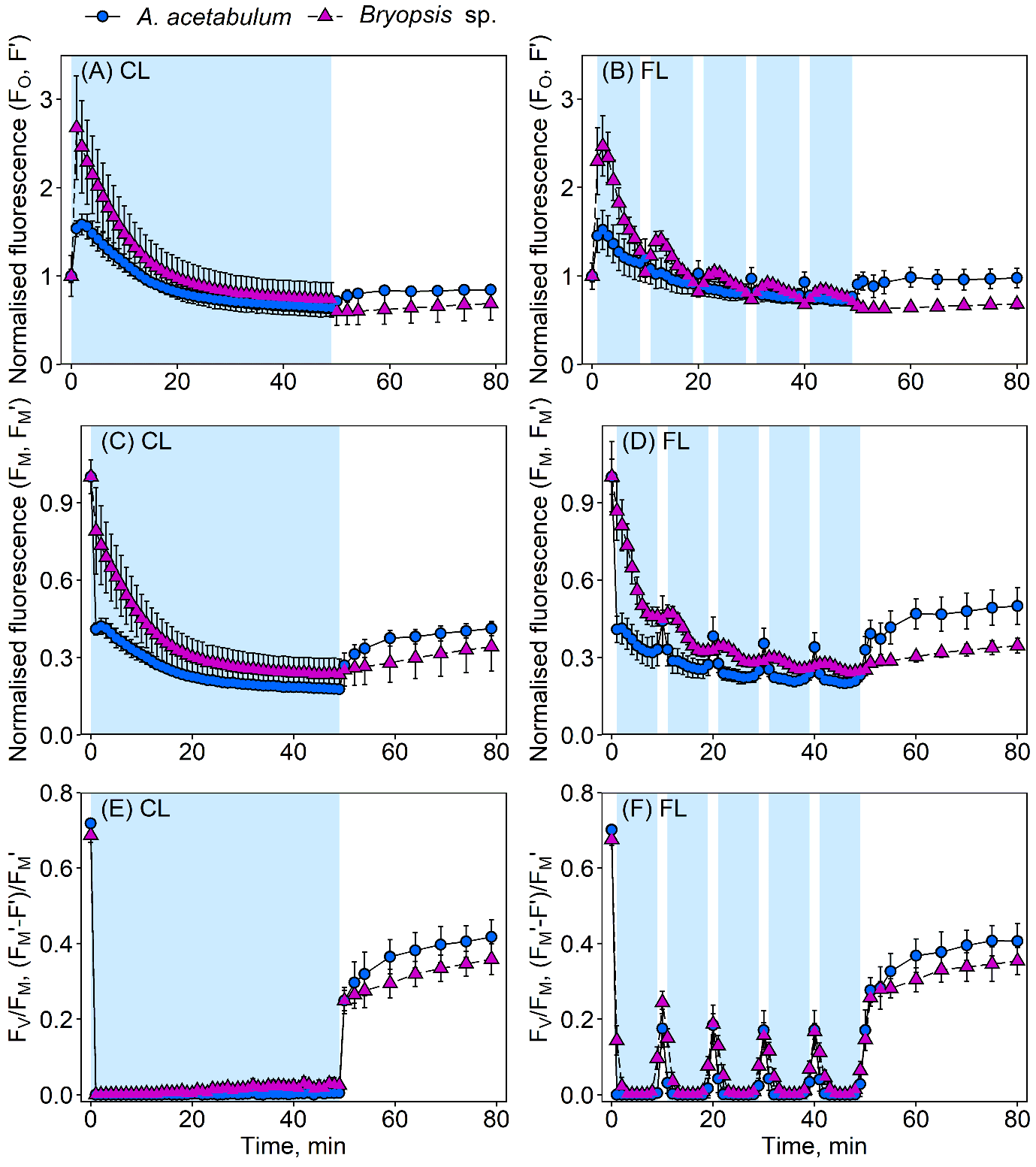


**Fig. S4**. Kinetics of chlorophyll *a* fluorescence parameters under constant and fluctuating light in *Acetabularia acetabulum* (blue circles) and *Bryopsis* sp. (purple triangles). Dark-acclimated algae were given a 50-min treatment with constant (A, C and E; PPFD 500 µmol m^-2^ s^-1^) or fluctuating (B, D and F; PPFD of 0 to 1000 µmol m^-2^ s^-1^; see Fig. S2) blue light (illustrated by the light blue panels) and subsequent dark incubation (up to 30 min, as indicated). (A and B) Minimum chlorophyll *a* fluorescence yield of a dark-acclimated sample (F_O_) or incident fluorescence yield under illumination (F'), normalised to the starting values, (C and D) maximum chlorophyll *a* fluorescence yield during a saturating pulse of a dark-acclimated sample (F_M_) or under light (F_M_'), normalised to the starting values, and (E and F) PSII activity of a dark-acclimated sample (F_V_/F_M_) or under light ((F_M_'-F')/F_M_'). All the treatments were conducted at room temperature in artificial seawater. Symbols show averages and error bars standard deviations, calculated based on three to six biological replicates.

**
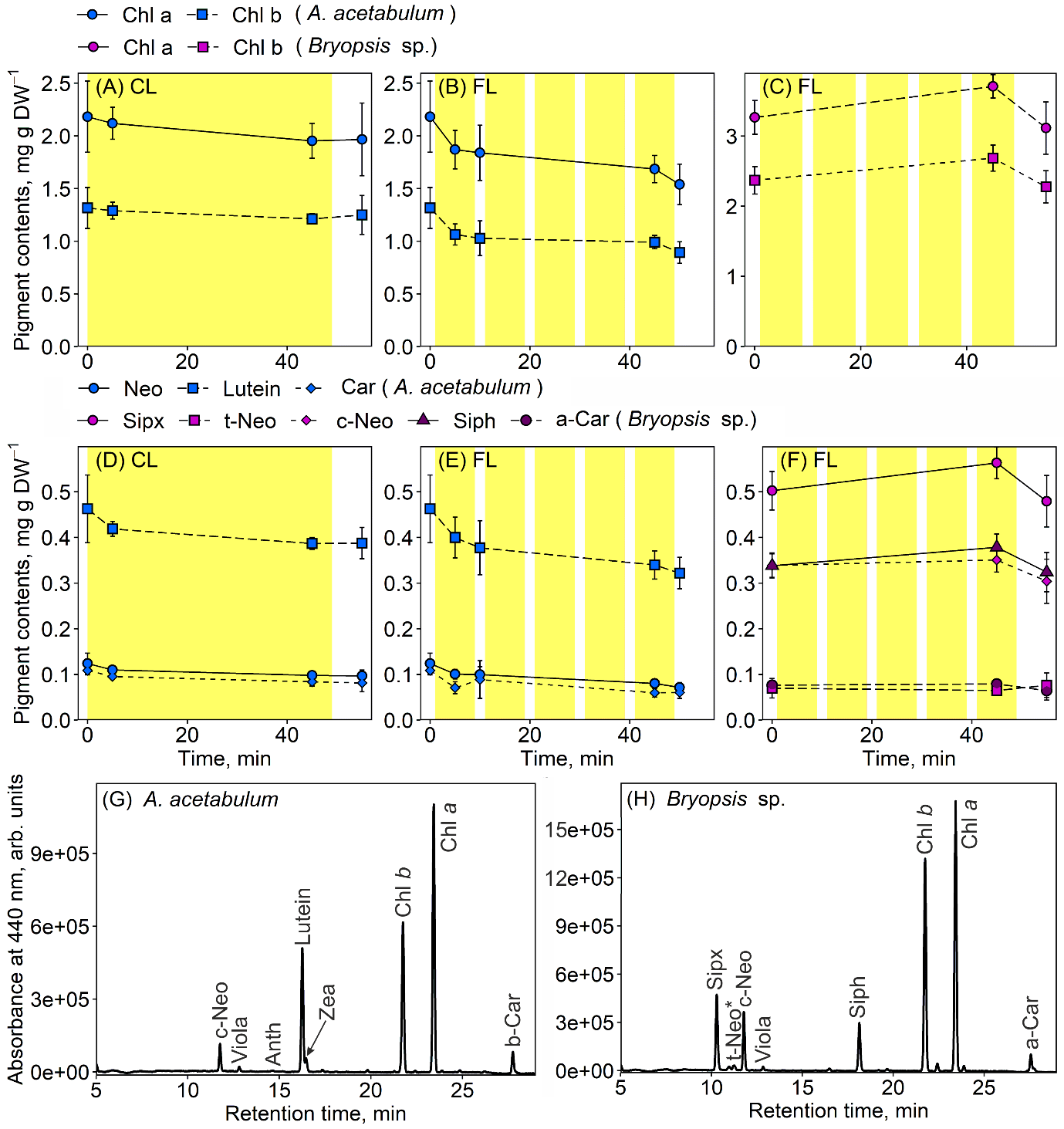
**

**Fig. S5**. Pigment contents under constant (A and D) and fluctuating (B, C, E–H) light in *Acetabularia acetabulum* (A, B, D, E and G; blue symbols) and *Bryopsis* sp. (C, F and H; purple symbols), quantified with HPLC. Dark acclimated algae were illuminated for 50 min with constant (PPFD 500 µmol m^-2^ s^-1^) or fluctuating (PPFD of 0 to 1000 µmol m^-2^ s^-1^; see Fig. S2) white light (illustrated by the yellow panels) and, in the case of constant light, incubated subsequently in darkness for five min. Contents of (A–C) chlorophylls *a* and *b*, (D and E) 9'-cis-neoxanthin (Neo), lutein (Lutein) and β,β-carotene (Car) in *A. acetabulum* and (F) siphonaxanthin (Sipx), all-trans-neoxanthin (t-Neo), 9'-cis-neoxanthin (c-Neo), siphonein (Siph) and β,ε-carotene (α-carotene; a-Car) in *Bryopsis* sp., quantified on dry weight (DW) basis. All the treatments were conducted at room temperature in artificial seawater. The symbols show averages and error bars standard deviations, calculated based on four biological replicates. Exemplary chromatograms at 440 nm, measured from of *A. acetabulum* (G) and *Bryopsis* sp. (H) after 45 min of the fluctuating light treatment. Identified photosynthetic pigments: 9'-cis-neoxanthin (c-Neo), violaxanthin (Viola), antheraxanthin (Anth), lutein (Lutein), zeaxanthin (Zea), chlorophyll *b* (Chl *b*), chlorophyll *a* (Chl *a*), β,β-carotene (b-Car), siphonaxanthin (Sipx), all-trans-neoxanthin (t-Neo), siphonein (Siph) and β,ε-carotene (a-Car). The asterisk after t-Neo indicates asymmetric peak and noisy absorption spectrum, suggesting co-elution with another pigment or a contaminant.


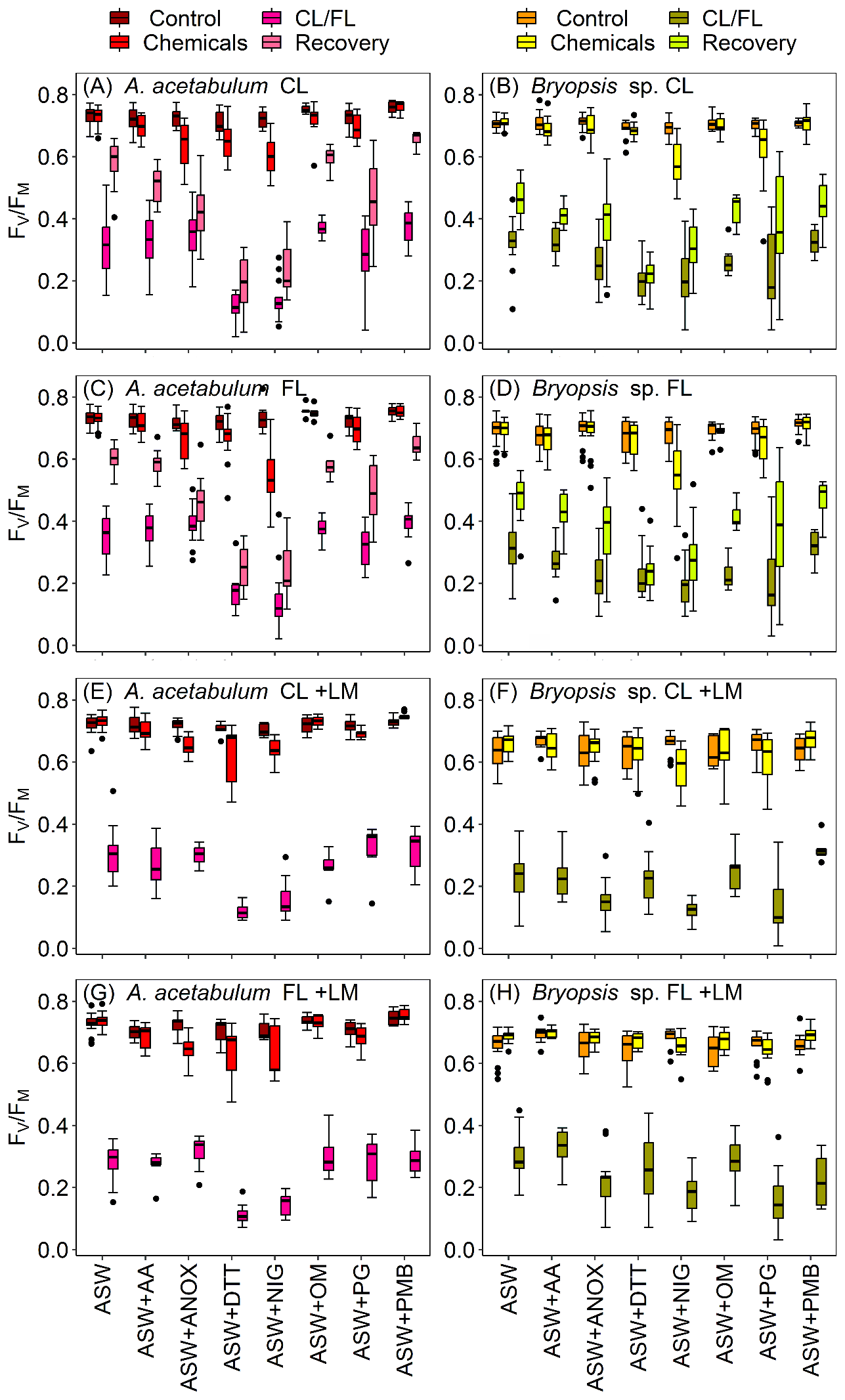


**Fig. S6**. PSII photoinhibition and recovery in *Acetabularia acetabulum* (A, C, E and G) and *Bryopsis* sp. (B, D, F and H). PSII activity was assayed with chlorophyll *a* fluorescence parameter F_V_/F_M_ from dark acclimated algae (Control), as well as after further 20 min incubation in darkness after addition of the indicated chemicals (Chemicals) or in plain artificial seawater (ASW), after 50 min illumination with constant (CL; white light of PPFD 500 µmol m^-2^ s^-1^) or fluctuating (FL; white light of PPFD 0 to 1000 µmol m^-2^ s^-1^; see Fig. S2) white light and subsequent 20-min dark incubation and finally, after two-hour recovery at low light (PPFD 10–20 µmol m^-2^ s^-1^) and subsequent 20-min dark incubation (Recovery). The added chemicals include either antimycin A (AA), glucose, glucose oxidase and catalase to induce anaerobicity (ANOX; see Fig. S9), dithiothreitol (DTT), nigericin (NIG), oligomycin (OM), propyl gallate (PG) or polymyxin B (PMB), as indicated. The high light treatments were also conducted with algae pre-incubated overnight in darkness in the presence of lincomycin (LM; E–H). All treatments were conducted at room temperature. The box plots show medians, 2^nd^ and 3^rd^ quartiles, error bars show minimum and maximum values and dots show outliers (> 1.5 times the interquartile range), calculated based on 6–28 biological replicates.


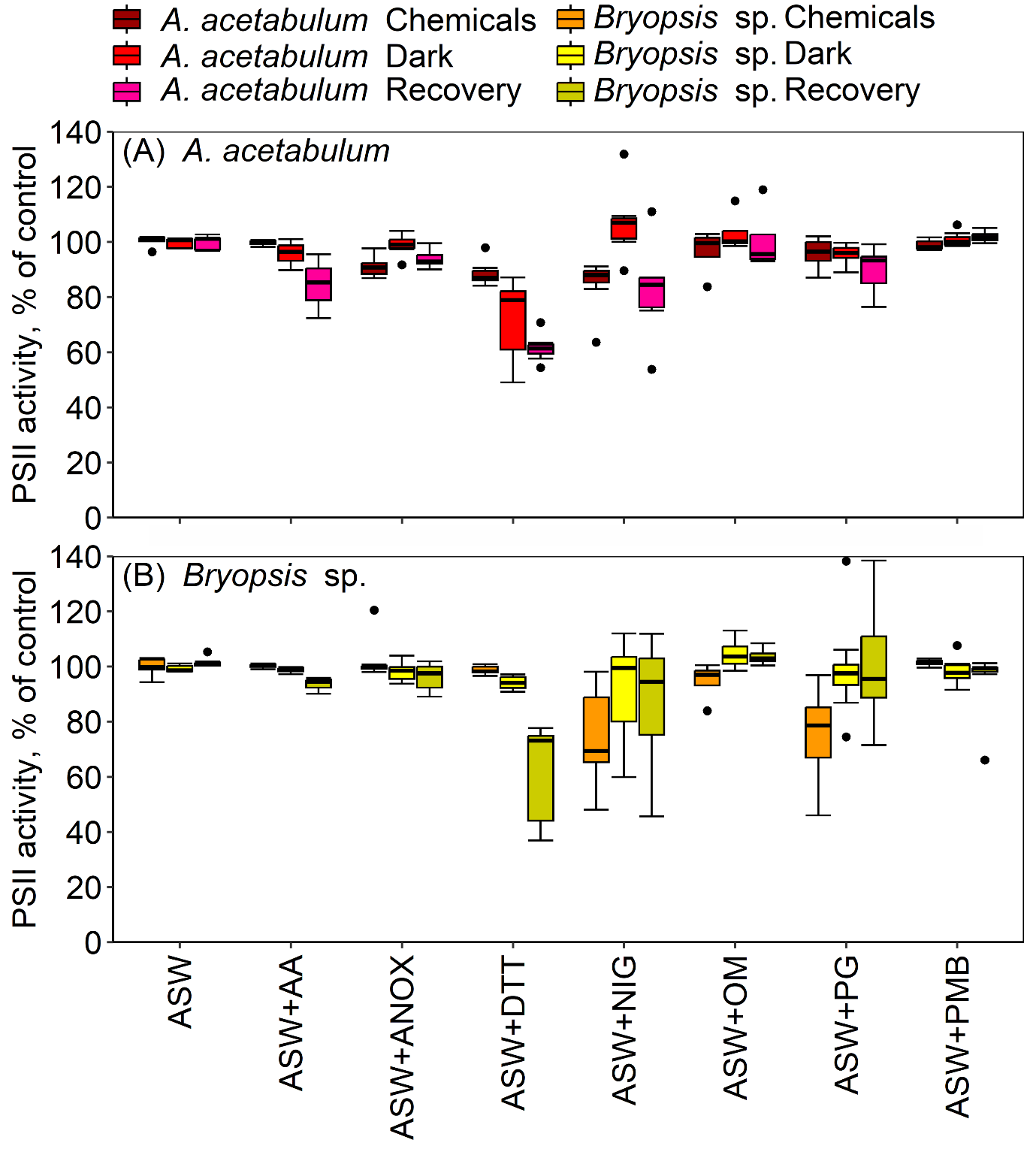


**Fig. S7**. Effects of different chemicals on PSII activity in darkness in *Acetabularia acetabulum* (A) and *Bryopsis* sp. (B). The algae were incubated in the dark with the chemicals for 20 min (Chemicals) or 90 min (Dark), after which they were let to recover at low light (PPFD of 10–20 µmol m^-2^ s^-1^) for two hours and again dark acclimated for 20 min (Recovery). All the treatments were conducted at room temperature in artificial seawater (ASW) supplemented with the following chemicals: either AA (antimycin A), anoxia (ANOX; glucose, glucose oxidase and catalase; to induce anaerobicity; Fig. S9), DTT (dithiothreitol), NIG (nigericin), OM (oligomycin), PG (propyl gallate) or PMB (polymyxin B). PSII activity was quantified with the chlorophyll *a* fluorescence parameter F_V_/F_M_. Control refers to the F_V_/F_M_ values of each sample prior to the addition of the chemicals (Chemicals) or after addition of the chemicals (Dark and Recovery). The box plots show medians, 2^nd^ and 3^rd^ quartiles, error bars show minimum and maximum values and dots show outliers (> 1.5 times the interquartile range), calculated based on three to eight biological replicates.


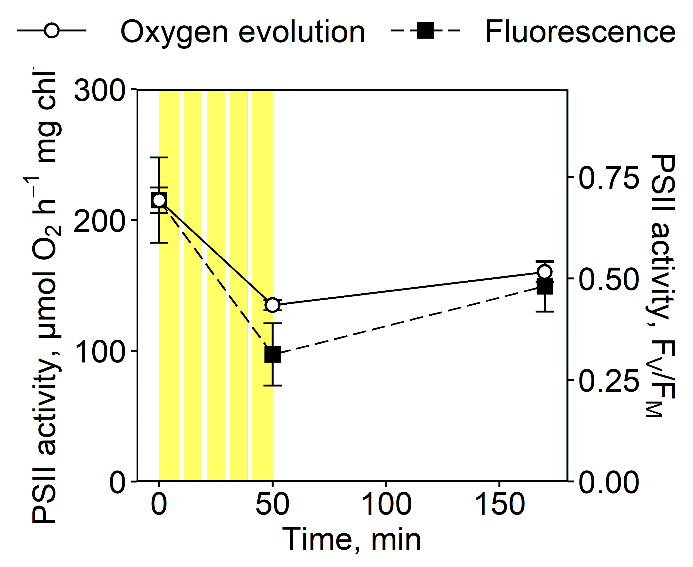


**Fig. S8**. PSII photoinhibition and recovery in *Bryopsis* sp., assayed with PSII oxygen evolution (open circles and solid lines), compared to a similar experiment where photoinhibition was assayed with a chlorophyll *a* fluorescence parameter F_V_/F_M_ (black squares and dashed lines; for the original data, see Figs 2 and S6). The algae were illuminated for 50 min with fluctuating white light (illustrated with the yellow panels; PPFD of 0 to 1000 µmol m^-2^ s^-1^; Fig. S2) and let to recover for two hours at low light (PPFD 10–20 µmol m^-2^ s^-1^). All the treatments were conducted at room temperature in artificial seawater. Thylakoid membranes were isolated before and after the high light treatment and after the recovery period. Immediately after the isolations, maximum rates of oxygen evolution by PSII were measured from the thylakoids under saturating light (PPFD 4000 µmol m^-2^ s^-1^), in the presence of artificial electron acceptors and an uncoupler of the proton gradient; the rates were quantified based on chlorophyll (chl) content of the sample. The symbols show averages and error bars standard deviations calculated based on three (oxygen evolution data) biological replicates (separate isolations).

**
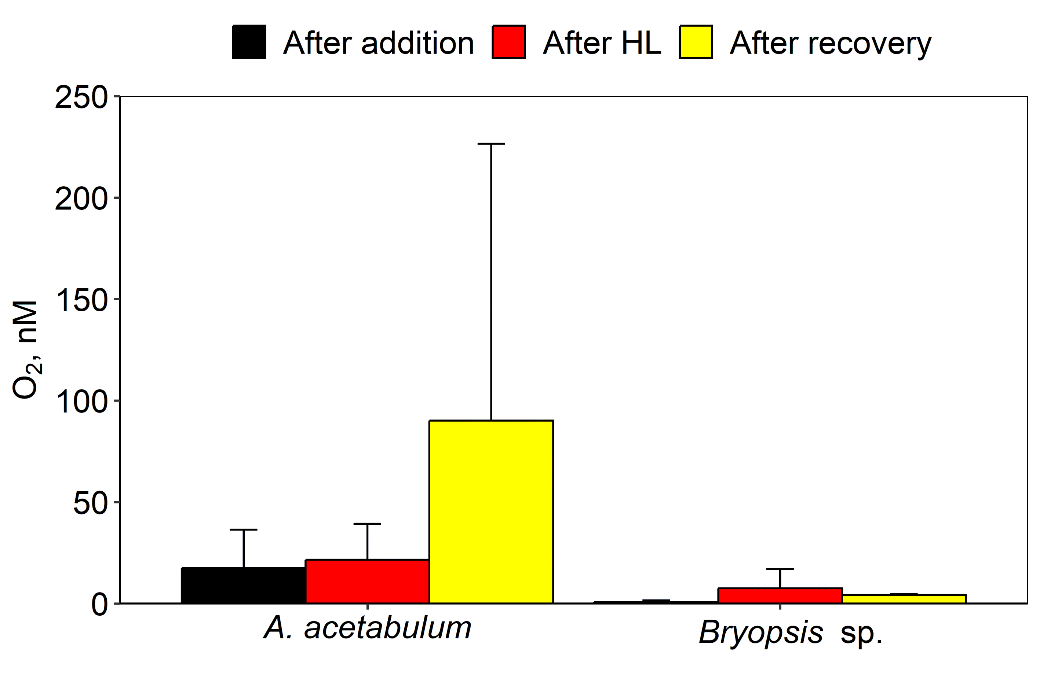
**

**Fig. S9**. Oxygen concentration in *Acetabularia acetabulum* and *Bryopsis* sp. samples kept in two mL of artificial seawater in the presence of glucose, glucose oxidase and catalase, measured right after addition of the chemicals (black bars), after 50 min illumination with constant white high light (HL; red bars; PPFD 500 µmol m^-2^ s^-1^) and after subsequent two hours at low light (yellow bars; PPFD 10–20 µmol m^-2^ s^-1^). All the treatments were conducted at 20°C. The bars show averages and error bars standard deviations calculated based on three to four biological replicates.


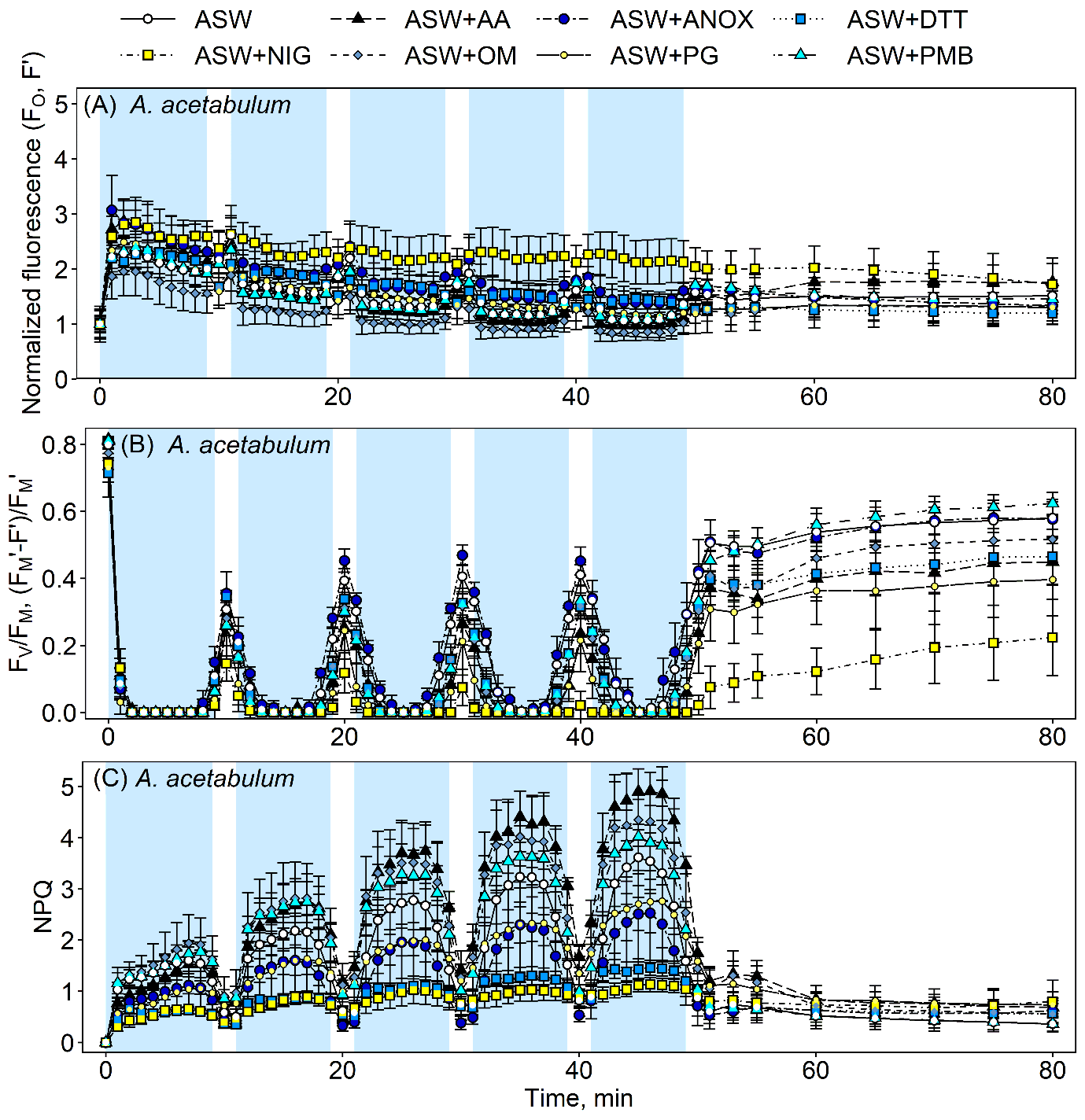


**Fig S10**. Chlorophyll *a* fluorescence kinetics under fluctuating light in *Acetabularia acetabulum*. Dark-acclimated algae were given a 50-min treatment with blue light (PPFD of 0 to 1000 µmol m^-2^ s^-1^; as illustrated by the light blue panels; Fig. S2) and subsequent 30-min darkness. The treatments were conducted at room temperature in artificial seawater (ASW) supplemented with the following chemicals: either AA (antimycin A), ANOX (glucose, glucose oxidase and catalase; to induce anaerobicity; Fig. S9), DTT (dithiothreitol), NIG (nigericin), OM (oligomycin), PG (propyl gallate) or PMB (polymyxin B), as indicated. (A) Minimum chlorophyll *a* fluorescence yield of a dark-acclimated sample (F_O_) or incident fluorescence yield under illumination (F'), normalised to the starting values, (B) PSII activity of a dark-acclimated sample (F_V_/F_M_) or under light ((F_M_'-F')/F_M_') and (C) non-photochemical quenching (NPQ). Symbols show averages and error bars standard deviations, calculated based on four to 12 biological replicates.


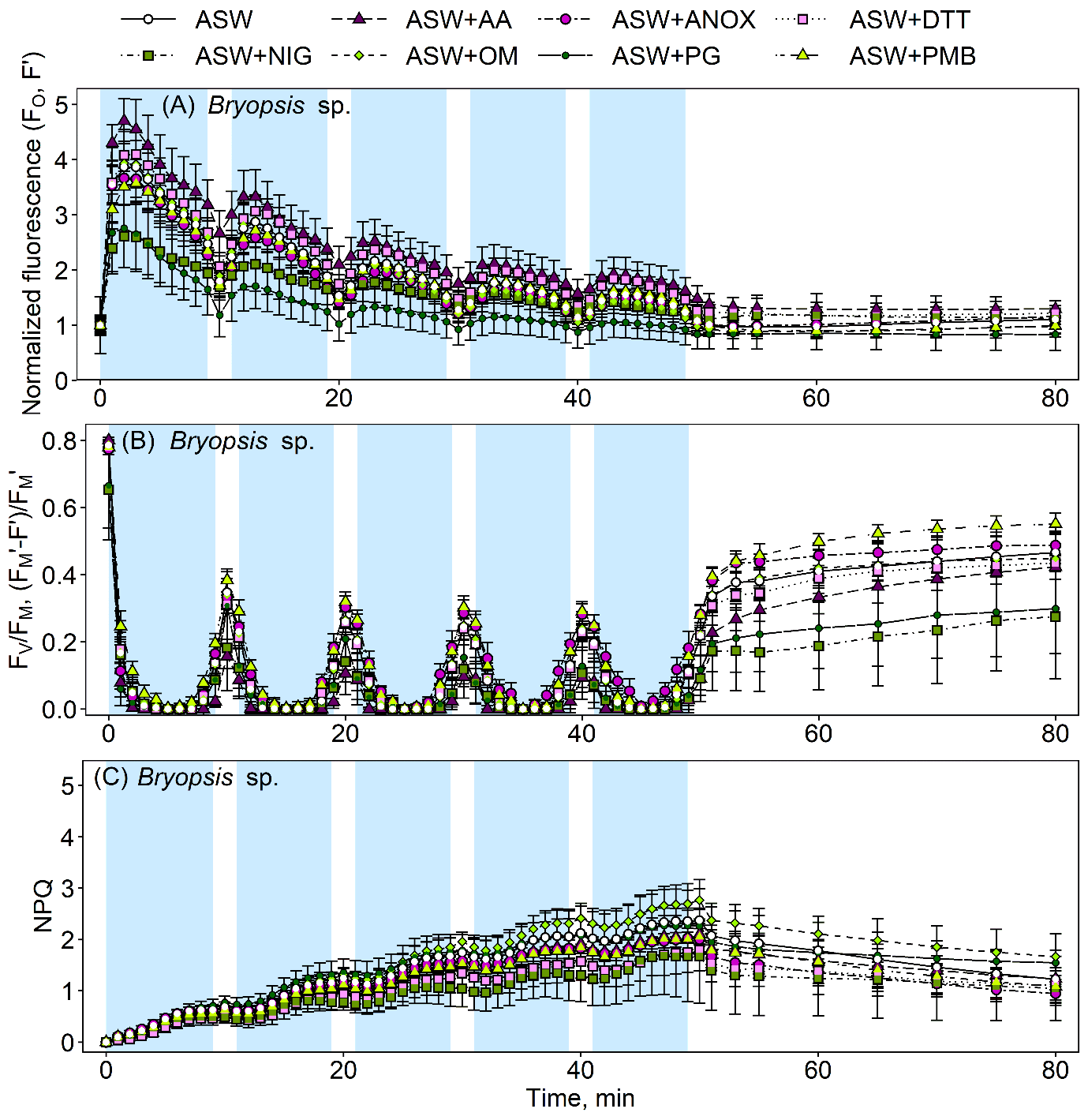


**Fig. S11**. Chlorophyll *a* fluorescence kinetics under fluctuating light in *Bryopsis* sp.. Dark-acclimated algae were given a 50-min treatment with blue light (PPFD of 0 to 1000 µmol m^-2^ s^-1^; as illustrated by the light blue panels; Fig. S2) and subsequent 30-min darkness. The treatments were conducted at room temperature in artificial seawater (ASW) supplemented with the following chemicals: either AA (antimycin A), ANOX (glucose, glucose oxidase and catalase; to induce anaerobicity; Fig. S9), DTT (dithiothreitol), NIG (nigericin), OM (oligomycin), PG (propyl gallate) or PMB (polymyxin B), as indicated. (A) Minimum chlorophyll *a* fluorescence yield of a dark-acclimated sample (F_O_) or incident fluorescence yield under illumination (F'), normalised to the starting values, (B) PSII activity of a dark-acclimated sample (F_V_/F_M_) or under light ((F_M_'-F')/F_M_') and (C) non-photochemical quenching (NPQ). Symbols show averages and error bars standard deviations, calculated based on four to 12 biological replicates.


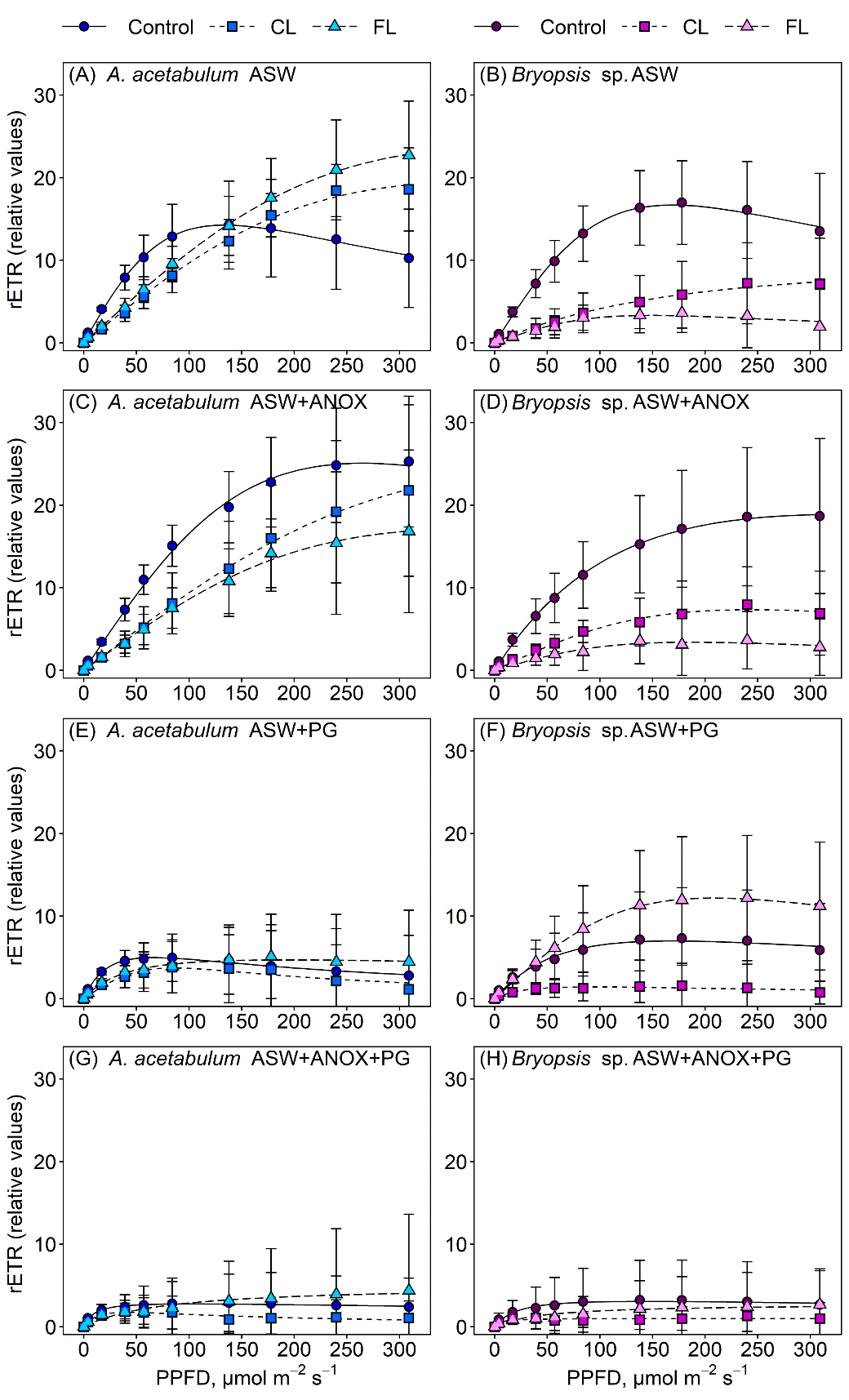


**Fig. S12**. Rapid light response curves, measured with chlorophyll *a* fluorescence from light-acclimated *Acetabularia acetabulum* and *Bryopsis* sp., either before (circles; Control) or after a 50-min illumination at room temperature in artificial seawater (ASW) with constant (squares; CL; PPFD 500 µmol m^-2^ s^-1^) or fluctuating (triangles; FL; PPFD of 0 to 1000 µmol m^-2^ s^-1^; Fig. S2) white light. During the light curve measurements, algae were kept in ASW (A, B), supplemented with glucose, glucose oxidase and catalase (C, D; ANOX; to induce anaerobicity; Fig. S9), propyl gallate (G, F; PG) or both (E, F), as indicated. The algae were illuminated for 60 s with increasing intensities of blue light (as indicated), after which a saturating light pulse was fired to calculate relative rates of electron transfer (rETR), as (F_M_'-F')/F_M_' x 0.5 x 0.84 x PPFD. Symbols show averages and error bars standard deviations calculated based on eight to 16 biological replications. Lines show best fits to the light response curves, modelled according to Eilers and Peeters (1988).


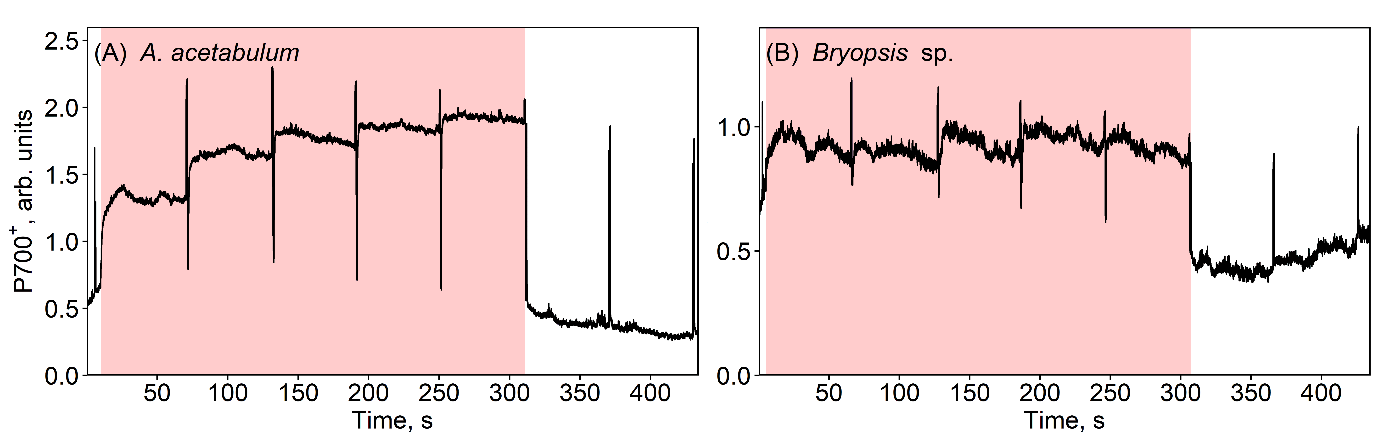


**Fig. S13**. Exemplary P700 redox kinetics in (A) *Acetabularia acetabulum* and (B) *Bryopsis* sp.. P700 oxidation (P700^+^; arbitrary units) under increasing intensities of red light (PPFDs 50, 110, 215, 550 and 1080 µmol m^-2^ s^-1^; 1 min with each intensity; indicated with the light red panel) and during subsequent two-min darkness was measured from dark acclimated algae in artificial seawater. Saturating pulses were fired at the end of each light intensity. All treatments were conducted under room temperature. Lines show individual measurements.

**Supplementary tables**

**Table S1**. Statistical significances of differences in photoinhibition (without and with lincomycin) and recovery between *Acetabularia acetabulum* illuminated under constant light in plain artificial seawater and the indicated treatments. See Figs 2 and S6 for the data. The significances have been obtained with heteroscedastic t-tests, using Bonferroni corrections.

| **Comparisons** | **Photoinhibition** | **Recovery** | **Photoinhibition +LM** |
| --- | --- | --- | --- |
| ASW vs chemicals (CL): |  |  |  |
| ASW vs ASW + AA | 0.0291 | **0.0004**** | 0.7717 |
| ASW vs ASW + ANOX | **0.0014*** | **2.7E-8***** | 0.1096 |
| ASW vs ASW + DTT | 0.3599 | **6.6E-15***** | 0.0976 |
| ASW vs ASW + NIG | **3.6E-9***** | **0.0014*** | **0.0016*** |
| ASW vs ASW + OM | 0.1091 | 0.4665 | 0.0071^•^ |
| ASW vs ASW + PG | 0.8374 | **0.0028*** | 0.0764 |
| ASW vs ASW + PMB | 0.2078 | 0.6373 | 0.6310 |
| No LM vs LM (ASW, CL) | 0.7982 | n.m. | - |
| CL vs FL (ASW) | 0.0910 | 0.3096 | 0.0580 |
| *A. acetabulum* vs *Bryopsis* sp. (ASW, CL) | 0.2399 | **2.4E-8***** | 0.1237 |

^•^ = P<0.1 (Bonferroni corrected value for 10 comparisons 0.01); * = P<0.05 (Bonferroni corrected value for 10 comparisons 0.005); ** = P<0.01 (Bonferroni corrected value for 10 comparisons 0.001); *** = P<0.001 (Bonferroni corrected value for 10 comparisons 0.0001). ASW = artificial seawater, AA = antimycin A, ANOX = anaerobicity, DTT = dithiothreitol, NIG = nigericin, OM = oligomycin, PG = propyl gallate, PMB = polymyxin B, LM = lincomycin, CL = constant light, FL = fluctuating light, n.m. = not measured.

**Table S2**. Statistical significances of differences in photoinhibition (without and with lincomycin) and recovery between *Acetabularia acetabulum* illuminated under fluctuating light in plain artificial seawater and the indicated treatments. See Figs 2 and S6 for the data. The significances have been obtained with heteroscedastic t-tests, using Bonferroni corrections.

| **Comparisons** | **Photoinhibition** | **Recovery** | **Photoinhibition +LM** |
| --- | --- | --- | --- |
| ASW vs chemicals (FL): |  |  |  |
| ASW vs ASW + AA | 0.0152 | **0.001**** | 0.5796 |
| ASW vs ASW + ANOX | **0.0045*** | **9.0E-6***** | **0.0008**** |
| ASW vs ASW + DTT | 0.0588 | **3.1E-13***** | 0.0096^•^ |
| ASW vs ASW + NIG | **0.0001**** | 0.0867 | **3.2E-6***** |
| ASW vs ASW + OM | 0.5626 | 0.2221 | 0.9543 |
| ASW vs ASW + PG | 0.5065 | **0.0013*** | 0.1655 |
| ASW vs ASW + PMB | 0.5229 | 0.7528 | 0.6849 |
| No LM vs LM (ASW, FL) | **0.0022*** | n.m. | - |
| CL vs FL (ASW) | See Table S1 | See Table S1 | See Table S1 |
| *A. acetabulum* vs *Bryopsis* sp. (ASW, FL) | 0.3297 | **2.2E-5***** | 0.0580 |

^•^ = P<0.1 (Bonferroni corrected value for 10 comparisons 0.01); * = P<0.05 (Bonferroni corrected value for 10 comparisons 0.005); ** = P<0.01 (Bonferroni corrected value for 10 comparisons 0.001); *** = P<0.001 (Bonferroni corrected value for 10 comparisons 0.0001). ASW = artificial seawater, AA = antimycin A, ANOX = anaerobicity, DTT = dithiothreitol, NIG = nigericin, OM = oligomycin, PG = propyl gallate, PMB = polymyxin B, LM = lincomycin, CL = constant light, FL = fluctuating light, n.m. = not measured.

**Table S3**. Statistical significances of differences in photoinhibition (without and with lincomycin) and recovery between *Bryopsis* sp. illuminated under constant light in plain artificial seawater and the indicated treatments. See Figs 2 and S6 for the data. The significances have been obtained with heteroscedastic t-tests, using Bonferroni corrections.

| **Comparisons** | **Photoinhibition** | **Recovery** | **Photoinhibition +LM** |
| --- | --- | --- | --- |
| ASW vs chemicals (CL): |  |  |  |
| ASW vs ASW + AA | 0.6301 | 0.1274 | 0.3987 |
| ASW vs ASW + ANOX | 0.0199 | 0.7495 | **0.0038*** |
| ASW vs ASW + DTT | **0.0023*** | **4.4E-7***** | 0.1080 |
| ASW vs ASW + NIG | 0.8649 | 0.3398 | 0.1530 |
| ASW vs ASW + OM | **0.0017*** | 0.1362 | 0.9947 |
| ASW vs ASW + PG | 0.0400 | 0.2980 | 0.0076^•^ |
| ASW vs ASW + PMB | 0.0054^•^ | 0.5795 | 0.0389 |
| No LM vs LM (ASW, CL) | 0.0106 | n.m. | - |
| CL vs FL (ASW) | 0.6581 | 0.0328 | 0.0450 |
| *A. acetabulum* vs *Bryopsis* sp. (ASW, CL) | See Table S1 | **See Table S1***** | See Table S1 |

^•^ = P<0.1 (Bonferroni corrected value for 10 comparisons 0.01); * = P<0.05 (Bonferroni corrected value for 10 comparisons 0.005); ** = P<0.01 (Bonferroni corrected value for 10 comparisons 0.001); *** = P<0.001 (Bonferroni corrected value for 10 comparisons 0.0001). ASW = artificial seawater, AA = antimycin A, ANOX = anaerobicity, DTT = dithiothreitol, NIG = nigericin, OM = oligomycin, PG = propyl gallate, PMB = polymyxin B, LM = lincomycin, CL = constant light, FL = fluctuating light, n.m. = not measured.

**Table S4**. Statistical significances of differences in photoinhibition (without and with lincomycin) and recovery between *Bryopsis* sp. illuminated under fluctuating light in plain artificial seawater and the indicated treatments. See Figs 2 and S6 for the data. The significances have been obtained with heteroscedastic t-tests, using Bonferroni corrections.

| **Comparisons** | **Photoinhibition** | **Recovery** | **Photoinhibition +LM** |
| --- | --- | --- | --- |
| ASW vs chemicals (FL): |  |  |  |
| ASW vs ASW + AA | 0.1668 | 0.9820 | 0.3842 |
| ASW vs ASW + ANOX | **0.0003**** | 0.1700 | 0.0265 |
| ASW vs ASW + DTT | 0.2745 | **8.0E-11***** | 0.9713 |
| ASW vs ASW + NIG | 0.6760 | 0.0092^•^ | 0.1061 |
| ASW vs ASW + OM | 0.0129 | 0.2966 | 0.4023 |
| ASW vs ASW + PG | **0.0044*** | 0.3726 | **3.6E-5***** |
| ASW vs ASW + PMB | 0.8475 | 0.8475 | 0.0266 |
| No LM vs LM (ASW, FL) | 0.6732 | n.m. | - |
| CL vs FL (ASW) | See Table S3 | See Table S3 | See Table S3 |
| *A. acetabulum* vs *Bryopsis* sp. (ASW, FL) | See Table S2 | **See Table S2**** | See Table S2 |

^•^ = P<0.1 (Bonferroni corrected value for 10 comparisons 0.01); * = P<0.05 (Bonferroni corrected value for 10 comparisons 0.005); ** = P<0.01 (Bonferroni corrected value for 10 comparisons 0.001); *** = P<0.001 (Bonferroni corrected value for 10 comparisons 0.0001). ASW = artificial seawater, AA = antimycin A, ANOX = anaerobicity, DTT = dithiothreitol, NIG = nigericin, OM = oligomycin, PG = propyl gallate, PMB = polymyxin B, LM = lincomycin, CL = constant light, FL = fluctuating light, n.m. = not measured.

**Table S5**. Statistical significances of differences in photochemical quenching (qP), maximum NPQ values and differences in incident fluorescence (F') between 12 and 13 min (low light) during fluctuating light treatment in *Acetabularia acetabulum* and *Bryopsis* sp. between illumination in plain artificial seawater and in the presence of the indicated chemicals. See Figs 4, S10 and S11 for the data. The significances have been obtained with heteroscedastic t-tests, using Bonferroni corrections.

| **Comparisons** | **qP** | **Maximum NPQ** | **Change in F**' **during low light** |
| --- | --- | --- | --- |
| *A. acetabulum***:** |  |  |  |
| ASW vs ASW + AA | **0.0066*** | 0.0146 | 0.0386 |
| ASW vs ASW + ANOX | 0.6310 | **0.0026*** | **8.0E-5***** |
| ASW vs ASW + DTT | 0.0259 | **3.1E-6***** | **2.1E-5***** |
| ASW vs ASW + NIG | **2.2E-9***** | **8.4E-7***** | **6.5E-8***** |
| ASW vs ASW + OM | **2.6E-5***** | 0.0313 | 0.1123 |
| ASW vs ASW + PG | **0.0001***** | 0.1037 | 1.5E-6 |
| ASW vs ASW + PMB | **5.6E-5***** | 0.2188 | 0.2628 |
| *Bryopsis* sp.: |  |  |  |
| ASW vs ASW + AA | **1.6E-5***** | 0.2679 | **0.0036*** |
| ASW vs ASW + ANOX | 0.0166 | 0.2051 | 0.0979 |
| ASW vs ASW + DTT | 0.2460 | 0.0863 | 0.2270 |
| ASW vs ASW + NIG | 0.0118 | 0.2680 | 0.0151 |
| ASW vs ASW + OM | 0.6947 | 0.1730 | 0.6653 |
| ASW vs ASW + PG | 0.0162 | 0.7599 | **0.0012**** |
| ASW vs ASW + PMB | 0.0360 | 0.2102 | 0.4672 |

^•^ = P<0.1 (Bonferroni corrected value for 7 comparisons 0.0143); * = P<0.05 (Bonferroni corrected value for 7 comparisons 0.0071); ** = P<0.01 (Bonferroni corrected value for 7 comparisons 0.0014); *** = P<0.001 (Bonferroni corrected value for 7 comparisons 0.00014). ASW = artificial seawater, AA = Antimycin A, ANOX = anaerobicity, DTT = dithiothreitol, NIG = nigericin, OM = oligomycin, PG = propyl gallate, PMB = polymyxin B.

**Table S6**. Statistical significances of differences in rapid light response curve parameters between non-illuminated and illuminated *Acetabularia acetabulum* and between the absence and presence of the indicated chemicals. See Figs 5 and S12 for the data. The significances have been obtained with heteroscedastic t-tests, using Bonferroni corrections.

| **Comparisons** | **Alpha** | **rETR_MAX_** | **I_K_** |
| --- | --- | --- | --- |
| ASW: |  |  |  |
| CTL vs CL | **9.0E-8***** | 0.0436 | 0.0069^•^ |
| CTL vs FL | **3.3E-7***** | 0.0089^•^ | **2.5E-6***** |
| ASW + ANOX: |  |  |  |
| CTL vs CL | **1.0E-8***** | 0.8511 | 0.0729 |
| CTL vs FL | **7.2E-9***** | 0.2124 | 0.0658 |
| ASW + PG: |  |  |  |
| CTL vs CL | **8.3E-6***** | 0.3881 | 0.3557 |
| CTL vs FL | **5.9E-6***** | 0.5161 | 0.1082 |
| ASW + PG + ANOX: |  |  |  |
| CTL vs CL | 0.0133 | 0.3221 | 0.3248 |
| CTL vs FL | **0.0014*** | 0.5107 | 0.2228 |
| ASW vs ASW + ANOX (CTL) | 0.5858 | **1.5E-5***** | **7.6E-5** |
| ASW vs ASW + PG (CTL) | 0.0498 | **0.0001**** | **1.1E-6** |
| ASW vs ASW + PG + ANOX (CTL) | 0.6456 | **5.1E-7***** | **0.0023*** |

^•^ = P<0.1 (Bonferroni corrected value for 12 comparisons 0.0091); * = P<0.05 (Bonferroni corrected value for 12 comparisons 0.0045); ** = P<0.01 (Bonferroni corrected value for 12 comparisons 0.0009); *** = P<0.001 (Bonferroni corrected value for 12 comparisons 9.1E-5). ASW = artificial seawater, ANOX = anaerobicity, PG = propyl gallate, CL = constant light, FL = fluctuating light, alpha = initial slope of a light response curve, rETR_MAX_ = a maximum rate of the electron transfer (relative values), I_K_ = saturating light intensity.

**Table S7**. Statistical significances of differences in rapid light curve parameters between non-illuminated and illuminated *Bryopsis* sp. and between in the absence and presence of the indicated chemicals. See Figs 5 and S12 for the data. The significances have been obtained with heteroscedastic t-tests, using Bonferroni corrections.

| **Comparisons** | **Alpha** | **rETR_MAX_** | **I_K_** |
| --- | --- | --- | --- |
| ASW: |  |  |  |
| CTL vs CL | **4.0E-8***** | 0.0915 | 0.1694 |
| CTL vs FL | **1.8E-9***** | **2.1E-5***** | 0.4865 |
| ASW + ANOX: |  |  |  |
| CTL vs CL | **3.4E-6***** | **0.0002**** | 0.9535 |
| CTL vs FL | **1.7E-6***** | **9.6E-6***** | 0.0211 |
| ASW + PG: |  |  |  |
| CTL vs CL | 0.0245 | **0.0008**** | 0.365 |
| CTL vs FL | **0.0025*** | 0.1627 | 0.0153 |
| ASW + PG + ANOX: |  |  |  |
| CTL vs CL | 0.0187 | 0.1134 | 0.5331 |
| CTL vs FL | 0.0149 | 0.5722 | 0.7490 |
| ASW vs ASW + ANOX (CTL) | 0.6727 | 0.1251 | 0.0994 |
| ASW vs ASW + PG (CTL) | 0.2963 | **4.9E-5***** | **0.0005**** |
| ASW vs ASW + PG + ANOX (CTL) | 0.6665 | **8.5E-8***** | 0.0179 |

^•^ = P<0.1 (Bonferroni corrected value for 11 comparisons 0.0091); * = P<0.05 (Bonferroni corrected value for 11 comparisons 0.0045); ** = P<0.01 (Bonferroni corrected value for 11 comparisons 0.0009); *** = P<0.001 (Bonferroni corrected value for 11 comparisons 9.1E-5). ASW = artificial seawater, ANOX = anaerobicity, PG = propyl gallate, CL = constant light, FL = fluctuating light, alpha = initial slope of a light response curve, ETR_MAX_ = a maximum rate of the electron transfer (relative values), I_K_ = saturating light intensity
